# A stromal β-defensin, DEFB124, contributes to cutaneous host defense against *Staphylococcus aureus*

**DOI:** 10.1016/j.isci.2026.117306

**Published:** 2026-08-27

**Authors:** Aaroh Joshi, Tomofumi Numata, Andrea Roso Mares, Hung Chan, Tatsuya Dokoshi, Anna Schwarz, Petra Kotzbeck, Jean-François Nicolas, Marc Vocanson, Peter Wolf, Vijaykumar Patra, Richard L. Gallo

**Affiliations:** 1 Department of Dermatology and Venereology, Medical University of Graz, 8010 Graz, Austria; 2 Department of Dermatology, University of California, San Diego, La Jolla, CA 92037, USA; 3 COREMED –Centre for Regenerative and Precision Medicine, Joanneum Research Forschungsgesellschaft mbH, Graz, Austria; 4 Research Unit for Tissue Repair and Reconstruction, Division of Plastic, Aesthetic and Reconstructive Surgery, Department of Surgery, Medical University of Graz, Graz, Austria; 5 CIRI, Centre International de Recherche en Infectiologie, INSERM, U1111, Université de Lyon 1, Ecole Normale Supérieure de Lyon, CNRS, UMR 5308, Lyon, France; 6 Allergy and Clinical Immunology Department, Lyon Sud University Hospital, Pierre-Bénite, Lyon, France

**Keywords:** antimicrobial peptides, defensins, atopic dermatitis, phototherapy, infection, *S. aureus*, stromal cells, fibroblasts, innate immunity

## Abstract

Antimicrobial peptides (AMPs) are key components of barrier immunity and are traditionally attributed to epithelial cells and granulocytes. Here, we report human β-defensin 124 (DEFB124) as a previously uncharacterized AMP predominantly produced by dermal stromal cells. Transcriptomic analyses across independent therapeutic cohorts showed consistent induction of DEFB124 during the restoration of skin homeostasis in atopic dermatitis (AD). Spatial transcriptomics, qPCR, and protein analyses localized DEFB124 expression to dermal fibroblasts and adipocytes. The murine ortholog β-defensin 25 (Defb25) showed a similar stromal expression pattern, was induced following intradermal *Staphylococcus aureus* (*S. aureus)* challenge, and was suppressed by type 2 cytokines through IL-4 receptor signaling. Recombinant DEFB124 showed dose-dependent antimicrobial activity *in vitro* and reduced bacterial burden *in vivo*, whereas Defb25 mRNA knockdown impaired fibroblast antimicrobial capacity and exacerbated *S. aureus* infection. These findings expand the known repertoire of cutaneous β-defensins and reveal stromal cells as an important source of cutaneous antimicrobial defense.

## Highlights

• DEFB124 is stromal-enriched, unlike largely keratinocyte-derived β-defensins in skin
• DEFB124 exhibits antimicrobial activity against clinically relevant bacterial pathogens
• Type 2 cytokines suppress DEFB124 expression, linking stromal immunity to atopic dermatitis
• Murine DEFB124 ortholog Defb25 protects against intradermal *S. aureus* infection

## Graphical abstract

**Figure:**
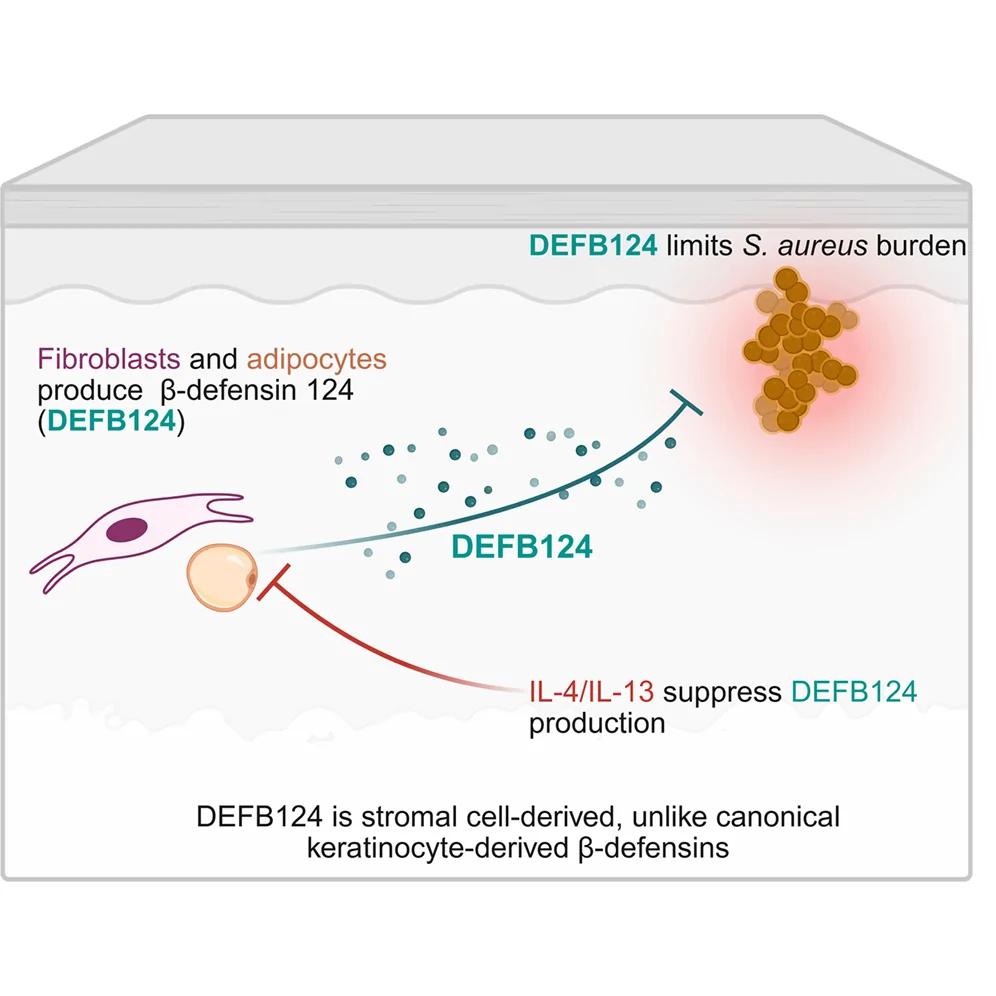

## Teaser

Biological sciences; Molecular biology; Immunology; Microbiology; Cell biology

## Introduction

Antimicrobial peptides (AMPs) are essential components of the innate immune system, providing the skin with rapid and broad-spectrum antimicrobial activity.^1^ They have been implicated in several diseases, highlighting their dual role in maintaining health and contributing to pathology.^2^^,^^3^^,^^4^ In the skin, these peptides are predominantly expressed by keratinocytes and myeloid cells and can be either constitutively active or induced by microbial and inflammatory stimuli. Among these AMPs, β-defensins constitute a prominent family of small cationic peptides with activity against bacteria, viruses, and fungi.^5^ In addition to their direct antimicrobial effects, β-defensins also function as immune modulators, influencing inflammatory responses and recruiting immune cells to sites of injury or infection.^6^^,^^7^^,^^8^

The human genome contains numerous β-defensin genes organized into clusters on several chromosomes, including chromosomes 8, 6, 20, and 11.^9^ The chromosome 8 cluster, which includes β-defensin 1 (DEFB1), β-defensin 2 (DEFB4), and β-defensin 3 (DEFB103), has been the primary focus of skin-related research.^10^^,^^11^ However, emerging evidence suggests that defensins encoded outside the canonical chromosome 8 cluster contribute to host defense and additional biological functions, although their roles in skin immunity, particularly with respect to their cellular sources and antimicrobial functions, remain poorly defined.^12^^,^^13^^,^^14^ Addressing this gap is essential for a more complete understanding of cutaneous host defense mechanisms.

In addition to their roles in infection, AMP changes have been linked to inflammatory skin diseases such as atopic dermatitis (AD). AD is a chronic inflammatory condition driven by type 2 immunity and elevated levels of cytokines such as IL-4 and IL-13, which can inhibit AMP production and impair skin barrier function.^15^^,^^16^ One hallmark of AD is the overgrowth of *Staphylococcus aureus* (*S. aureus*), which is commonly observed in lesional skin and is known to exacerbate disease by promoting inflammation, pruritus, and barrier disruption.^17^^,^^18^^,^^19^^,^^20^ Reduced expression of specific AMPs, including β-defensins with activity against *S. aureus*, is thought to contribute to this microbial imbalance, thereby compromising the skin’s innate immune barrier and facilitating *S. aureus* colonization and persistence.^21^^,^^22^^,^^23^

Here we identify β-defensin 124 (DEFB124), a defensin encoded within the chromosome 20 cluster, as a previously uncharacterized AMP expressed in human skin. We show that DEFB124 is predominantly produced by dermal stromal cells and contributes to cutaneous antimicrobial defense.

These findings expand the repertoire of defensins involved in skin immunity and highlight dermal stromal cells as an additional source of antimicrobial defense.

## Results

### Increased expression of DEFB124 in improving lesions of AD

**Figure 1:**
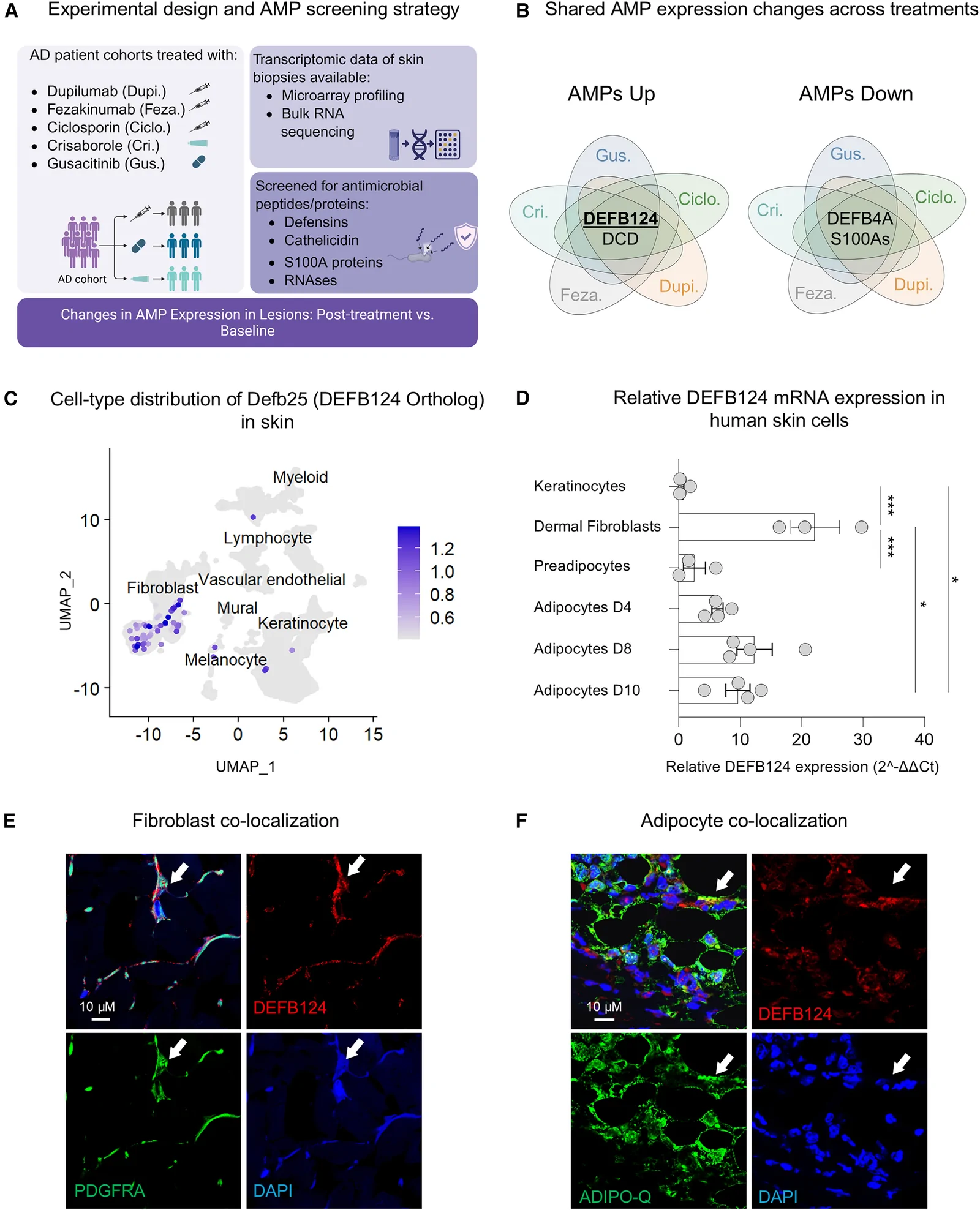
DEFB124 is expressed in skin and predominantly localized to stromal cells (A) Workflow for profiling antimicrobial gene expression in lesional AD skin pre- and post-treatment. Transcriptomic datasets from clinical studies involving AD patients treated with dupilumab, crisaborole, gusacitinib, fezakinumab, or ciclosporin were analyzed using either microarray or bulk RNA-seq. Samples were screened for expression of genes encoding antimicrobial peptides (AMPs), including defensins, cathelicidins, S100 proteins, and RNases. Differences in expression were calculated between improving lesions post-treatment and baseline lesional skin. See also Figure S1. (B) Venn diagrams showing overlap of upregulated (left) and downregulated (right) AMP genes across treatments. DEFB124 and DCD were consistently upregulated. In contrast, DEFB4A and members of the S100A family were commonly downregulated across treatments. (C) Cell type-specific expression of Defb25 in murine skin derived from single-cell RNA-seq datasets. Fibroblasts were identified as the predominant cellular source of Defb25 transcripts in the skin. See also Figures S2 and S3. (D) Relative DEFB124 mRNA expression across isolated human skin cell populations, including keratinocytes, dermal fibroblasts, preadipocytes, and adipocytes differentiated for 4 (D4), 8 (D8), or 10 (D10) days. Gene expression was analyzed by qPCR and normalized to GAPDH expression using the 2^∧^-ΔΔCt method. Data are presented as mean ± SEM. Each dot represents an individual donor-derived biological replicate (*n* = 3–4). Statistical significance was determined using one-way ANOVA; ∗*p* < 0.05, ∗∗*p* < 0.01, ∗∗∗*p* < 0.001. See also Figure S4. (E and F) Confocal immunofluorescence imaging of human dermis demonstrates co-localization of DEFB124 (red) with the fibroblast marker PDGFRA (green) (E) and Adipoq (F). Nuclei are counterstained with DAPI (blue). Arrows indicate double-positive cells. Scale bars, 10 μm. See also Figure S3C.

To identify AMPs potentially involved in the resolution of AD, we analyzed transcriptomic datasets (bulk RNA-seq or microarray) from published clinical studies, where AD patients were treated with either the topical agent crisaborole,^24^ the oral agent gusacitinib (ASN002),^25^, or systemic biologics, including dupilumab,^26^ cyclosporin,^26^ and fezakinumab^27^ (Figure 1A; Table S1). We focused on the expression profiles of key AMP gene families—defensins, cathelicidin, S100A proteins, and RNases. We compared post-treatment improving lesions to baseline (pre-treatment) skin samples.

Among these AMPs, DEFB124 and dermcidin (DCD) were consistently upregulated across all treatment groups, indicating a potential signature associated with lesion improvement (Figure 1B, left). In contrast, DEFB4A and S100A family genes were uniformly downregulated in improving lesions compared to baseline (Figure 1B, right).

To evaluate the baseline tissue distribution of DEFB124, we queried the Genevestigator database,^28^ which compiles normalized transcriptomic data from thousands of human samples. As shown in Figure S1A, DEFB124 is expressed across multiple tissues including testis, lung, nasal tissue, and colon with detectable and consistent expression in skin (*n* = 2,383), within a medium-to-high range relative to other epithelial tissues. Together, these findings suggest that DEFB124 transcripts are consistently expressed in the skin and may represent an AMP relevant to AD resolution.

### DEFB124 is predominantly expressed by stromal cells in human skin

To place these findings in an evolutionary context, we next examined mouse β-defensin 25 (Defb25), the putative ortholog of human DEFB124 based on conserved synteny (i.e., preservation of gene order across species) and sequence similarity within mammalian β-defensin gene clusters (Figure S1B).^29^ Sequence alignment revealed ∼71% identity, including conserved cysteine residues and high similarity across the mature peptide region (Figure S2A). Spatial transcriptomics of healthy mouse embryos localized Defb25 to stromal regions of multiple tissues including the skin (Figure S2B). Consistently, single-cell RNA sequencing (scRNA-seq) of adult mouse and human skin identified fibroblasts and pericytes as the predominant Defb25/DEFB124-expressing populations, followed by sebaceous and myeloid cells (Figures 1C and S2C).

Direct comparison with murine orthologs of human β-defensins, Defb14 (ortholog of human β-defensin 3), Defb1 (β-defensin 1), and Defb2 (β-defensin 2), revealed a distinct expression pattern compared to Defb25. While these established defensins were predominantly expressed in keratinocytes, Defb25 was primarily detected in fibroblasts (Figures S3A and S3B).

To further confirm the relative expression of DEFB124 across human skin cell types, we performed qPCR on RNA isolated from human keratinocytes, dermal fibroblasts, stromal preadipocytes, and differentiated adipocytes. DEFB124 expression was highest in fibroblasts, followed by adipocytes and preadipocytes, while keratinocytes exhibited minimal to absent expression. In contrast, DEFB1 expression was highest in keratinocytes (Figures 1D and S4).

To experimentally validate these findings at the protein level, we performed western blot analysis in a human fibroblast-like preadipocyte cell line. A distinct band at ∼5.2 kDa was detected, consistent with the predicted molecular weight of the mature DEFB124 peptide (Figure S2D). Immunofluorescence analysis of human dermis further confirmed DEFB124 expression in fibroblasts, as demonstrated by its colocalization with the canonical fibroblast markers PDGFRA and vimentin (Figures 1E and S3C, top). In addition, DEFB124-positive cells were detected in the basal layer of the epidermis. Co-immunostaining with MELAN-A identified these cells as melanocytes (Figure S3C, bottom).

In human dermis, immunostaining with the adipocyte marker adiponectin and FABP4 revealed strong DEFB124 expression in adipocytes under inflammatory conditions (Figures 1F and S3C, middle).

Together, these findings establish DEFB124/Defb25 as a conserved β-defensin predominantly expressed by stromal cell populations in skin.

### *S. aureus* infection induces Defb25, while type 2 cytokines suppress its expression via Il4-Rα

**Figure 2:**
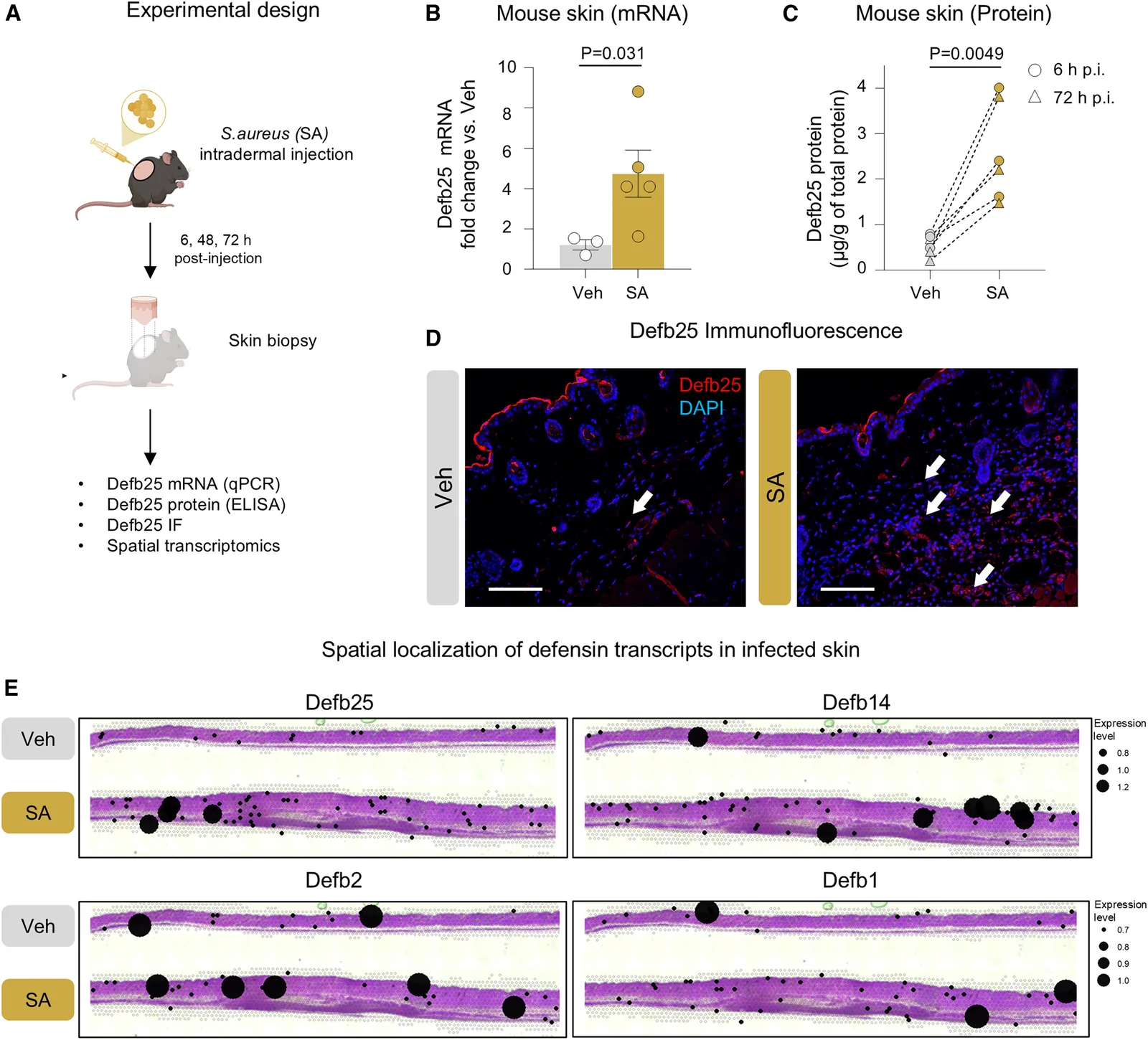
Regulation of Defb25 by *S. aureus* and AD-associated cytokines (A) Schematic of the *in vivo* experiment. Mice were intradermally injected with *S. aureus* USA300 (SA), and skin biopsies were collected 6, 48, or 72 h later for spatial transcriptomic analysis of Defb25 mRNA. (B) qPCR analysis of whole mouse skin confirms upregulation of Defb25 mRNA after *S. aureus* challenge. Data represent fold change relative to vehicle control (Mann-Whitney test, *p* = 0.031, *n* = 3–5 biological replicates). See also Figure S5A. (C) Quantification of Defb25 protein using enzyme-linked immunosorbent assay (ELISA) in mouse skin following *S. aureus* injection, measured at early (6 h) and later (72 h) time points post-infection, demonstrating increased protein levels compared with vehicle controls (paired *t* test, *n* = 3 biological replicates per time point). See also Figure S5B. (D) Immunofluorescence staining of mouse skin sections showing Defb25 protein localization following *S. aureus* infection. Defb25 is shown in red, and nuclei are counterstained with DAPI (blue). Compared with vehicle-treated skin, infected tissue shows increased Defb25 signal within the dermal compartment. Arrows indicate representative Defb25-positive cells. Scale bars, 100 μm. (E) Spatial transcriptomic analysis of defensin gene expression in mouse skin following *S. aureus* infection. Spatial feature maps display the distribution of Defb25, Defb14, Defb2, and Defb1 transcripts in skin sections from vehicle-treated (Veh.) and *S. aureus* (SA)-infected mice. Black spots represent spatially detected transcripts across the tissue, with spot size reflecting expression level.

β-Defensins play important roles in host defense during *S. aureus* infection, and *S. aureus* exhibits enhanced survival on AD skin, penetrates the dermis, and contributes to disease pathogenesis.^17^^,^^22^^,^^23^^,^^30^ To assess whether Defb25 expression is modulated by *S. aureus* challenge, we performed intradermal injections of *S. aureus* into the dorsal skin of mice and harvested tissue at defined points for analysis (Figures 2A and S5A). Spatial transcriptomic analysis revealed an expansion of Defb25-expressing regions in *S. aureus*-treated skin compared to vehicle controls (Figure 2E). In contrast to Defb25, other murine β-defensins (Defb14, Defb2, and Defb1) display distinct spatial expression patterns (Figure 2E), consistent with their predominantly epithelial localization. qPCR confirmed significant upregulation of Defb25 mRNA following infection (Figures 2A and S5A). Increased protein expression was further validated by immunofluorescence staining and ELISA of infected skin (Figures 2C and 2D). Post-infection, Defb25 protein expression levels were comparable to those of Defb14 (β-defensin 3 ortholog) (Figure S4B). Defb25 induction was detectable at both early (6 h) and later (72 h) time points post-infection (Figure S5A), indicating a sustained response to bacterial challenge.

**Figure 3:**
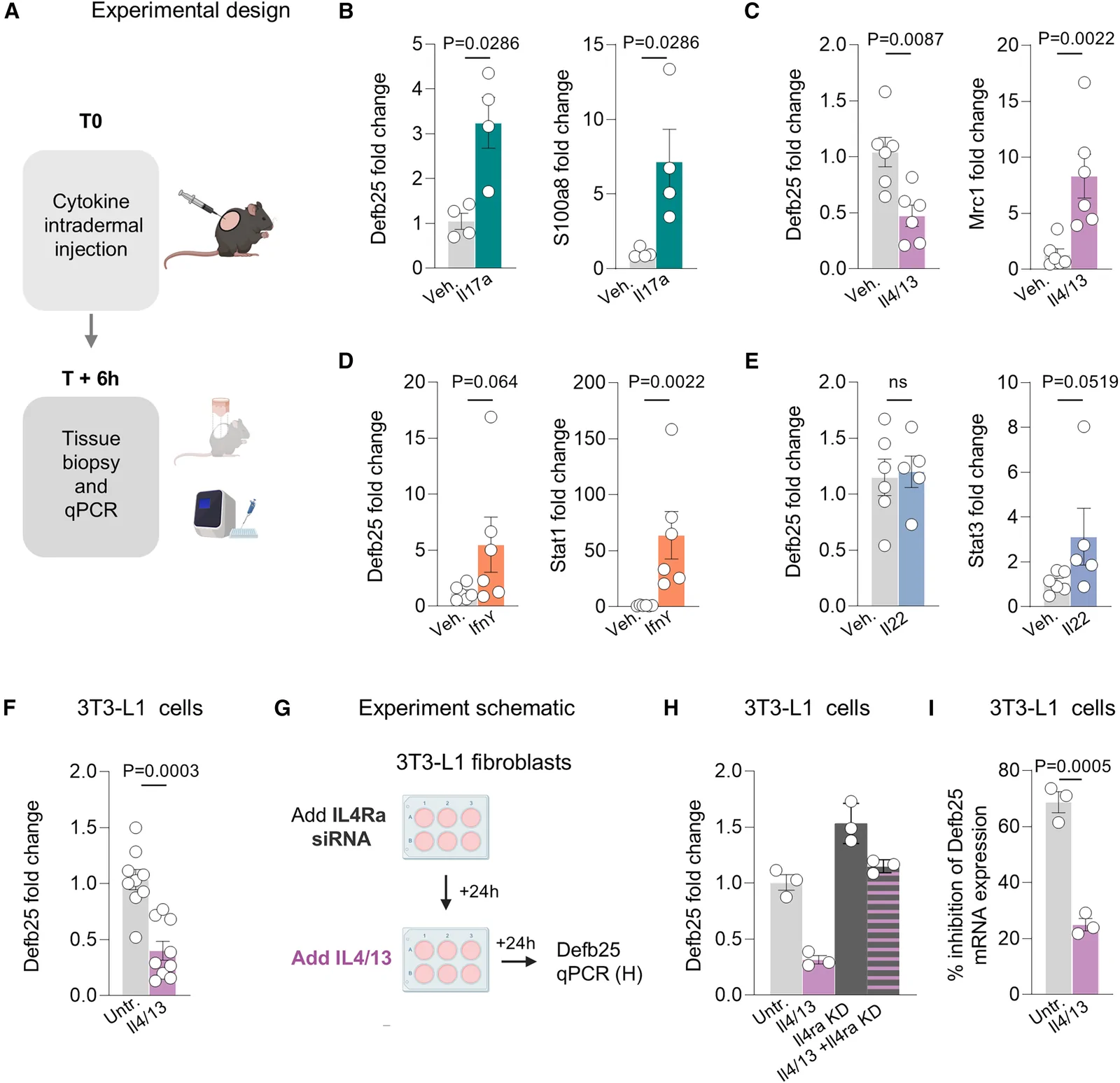
Regulation of Defb25 by cytokines (A–E) Cytokine-mediated regulation of Defb25 expression *in vivo*. Mice received intradermal injections of cytokines, and skin biopsies were collected 6 h post-injection for qPCR analysis. Fold changes in Defb25 and cytokine-responsive marker genes (S100a8, Mrc1, Stat1, and Stat3) are shown relative to vehicle-treated controls following stimulation with IL-17A (B), IL-4/13 (C), IFNγ (D), or IL-22 (E). Each point represents an individual mouse. Statistical significance was determined using the Mann-Whitney test, *n* = 4–6. See also Figure S5C. (F) qPCR analysis of Defb25 mRNA in 3T3-L1 fibroblasts following IL-4/13 stimulation, confirming cytokine-mediated suppression (Mann-Whitney tes*t, p* = 0.0003, *n* = 3). (G) Schematic of the *in vitro* IL-4Rα knockdown experiment. 3T3-L1 fibroblasts were transfected with IL-4Rα siRNA, followed by IL-4/13 stimulation and measurement of Defb25 expression by qPCR. (H) qPCR analysis of *Defb25* mRNA in 3T3-L1 fibroblasts treated with IL-4/13 in the presence or absence of IL-4Rα knockdown. Suppression of Defb25 by IL-4/13 is partially reversed upon IL-4Rα silencing. (I) Quantification of percent inhibition of Defb25 mRNA expression in 3T3-L1 fibroblasts. The graph shows the percentage reduction in expression relative to untreated controls, calculated from the fold-change values shown in H (*n* = 3).

We next investigated cytokine-mediated regulation of Defb25. *In vivo* cytokine administration demonstrated that IL-17A significantly increased Defb25 expression in mouse skin (Figure 3B), consistent with its role in antimicrobial defense pathways. IFNγ treatment showed a modest upward trend in Defb25 (Figure 3D, bottom left), and robust induction of the canonical IFNγ target gene *Stat1*, confirming effective pathway activation. IL-22 has been shown to induce defensin expression in epithelial cells. However, when we examined Defb25 mRNA levels, we did not observe a significant change in its expression following intradermal injection of murine IL-22 (Figure 3E).

In contrast, type 2 cytokines characteristic of AD had an opposing effect as *in vivo* IL-4/IL-13 administration showed reduced Defb25 expression levels in mouse skin (Figure 3B). Consistent with this observation, treatment of 3T3-L1 fibroblasts with IL-4 and IL-13 markedly suppressed Defb25 expression (Figure 3F).

To determine whether this suppression was mediated through IL-4 receptor α (IL-4Rα) signaling, fibroblasts were transfected with IL-4Rα siRNA prior to cytokine stimulation (Figures 3G–3I). Knockdown of IL-4Rα partially restored Defb25 expression in the presence of IL-4/IL-13 (Figure 3H), significantly attenuating the inhibitory effect (Figure 3I). These data demonstrate that type 2 cytokine-mediated repression of Defb25 is dependent, at least in part, on IL-4Rα signaling.

Collectively, these findings establish that Defb25 is dynamically regulated during cutaneous infection. Defb25 is robustly induced following *S. aureus* challenge and promoted by IL-17A-driven inflammatory signaling, supporting its role as a stromal antimicrobial effector. In contrast, type 2 cytokines characteristic of AD suppress Defb25 expression via IL-4Rα signaling. These findings suggest that bacterial infection induces Defb25 as part of dermal host defense, whereas type 2 inflammation suppresses this response, potentially contributing to enhanced *S. aureus* persistence in AD skin.

### DEFB124 shows defensin-like structural features and antimicrobial activity

Having demonstrated that Defb25 is induced during *S. aureus* infection and modulated by inflammatory cytokines (Figures 2 and 3), we next investigated whether DEFB124 possesses structural and functional properties consistent with a direct antimicrobial effector.

**Figure 4:**
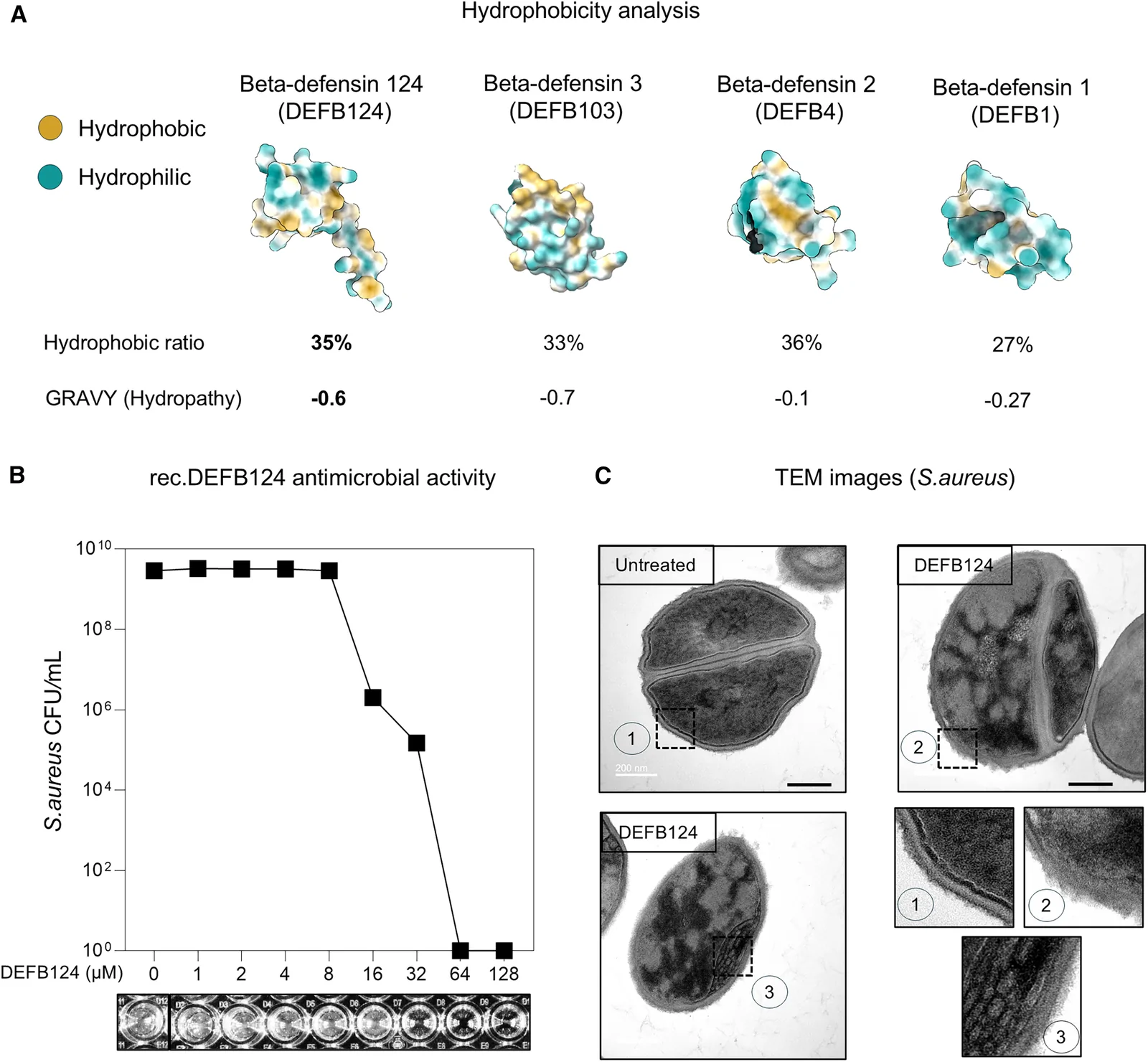
Structural features and anti-*S. aureus* activity of DEFB124 (A) Surface hydrophobicity analysis of DEFB124 compared to other skin-expressed β-defensins, including DEFB103A (β-defensin 3), DEFB4A (β-defensin 2), and DEFB1 (β-defensin 1). Protein structures were retrieved from UniProt and visualized in ChimeraX. Hydrophobic and hydrophilic residues are shown in gold and teal, respectively. DEFB124 exhibits a hydrophobic residue ratio of 35% and a GRAVY (grand average of hydropathy) score of -0.6, consistent with moderate hydrophilicity relative to other β-defensins. See also Figures S5D and S5E. (B) Antimicrobial activity of recombinant DEFB124 against *S. aureus*. Bacterial CFU/mL were quantified 24 h post-treatment with increasing concentrations of rec. DEFB124. DEFB124 exhibited potent, dose-dependent antibacterial activity. Bottom: well-based turbidity assay visualized 18 h post-incubation with rec. DEFB124 demonstrates visible inhibition of bacterial growth at higher concentrations. (C) Transmission electron microscopy (TEM) of *S. aureus* following DEFB124 treatment. Untreated bacteria (*S. aureus*) exhibit intact morphology, while DEFB124-treated cells display membrane disruption and formation of mesosome-like structures (insets 1, 2, and 3 respectively). Scale bars, 200 nm.

To explore its structural properties, we retrieved the DEFB124 peptide sequence from UniProt^31^ and generated 3D models using ChimeraX,^32^ comparing them with known defensins. The modeling revealed an amphipathic surface, with clear hydrophobic and hydrophilic regions, a hallmark of β-defensins (Figure 4A). Quantitative features supported this resemblance: the hydrophobic ratio (35%) and the grand average of hydropathy (GRAVY) score (-0.6), which ranges from -2 (hydrophilic) to +2 (hydrophobic), indicate an overall amphipathic character, closely matching that of DEFB103, a potent AMP. Electrostatic surface analysis showed that DEFB124 carries a mild net positive charge (+1.5), consistent with cationic AMP properties though lower than DEFB103 (Figure S5C). Finally, amino acid composition analysis highlighted enrichment in hydrophobic and flexible residues, along with a cysteine content comparable to that of skin-expressed defensins (Figure S5D). Together, these features suggest that DEFB124 shares the structural and biochemical hallmarks of functional antimicrobial peptides.

**Figure 5:**
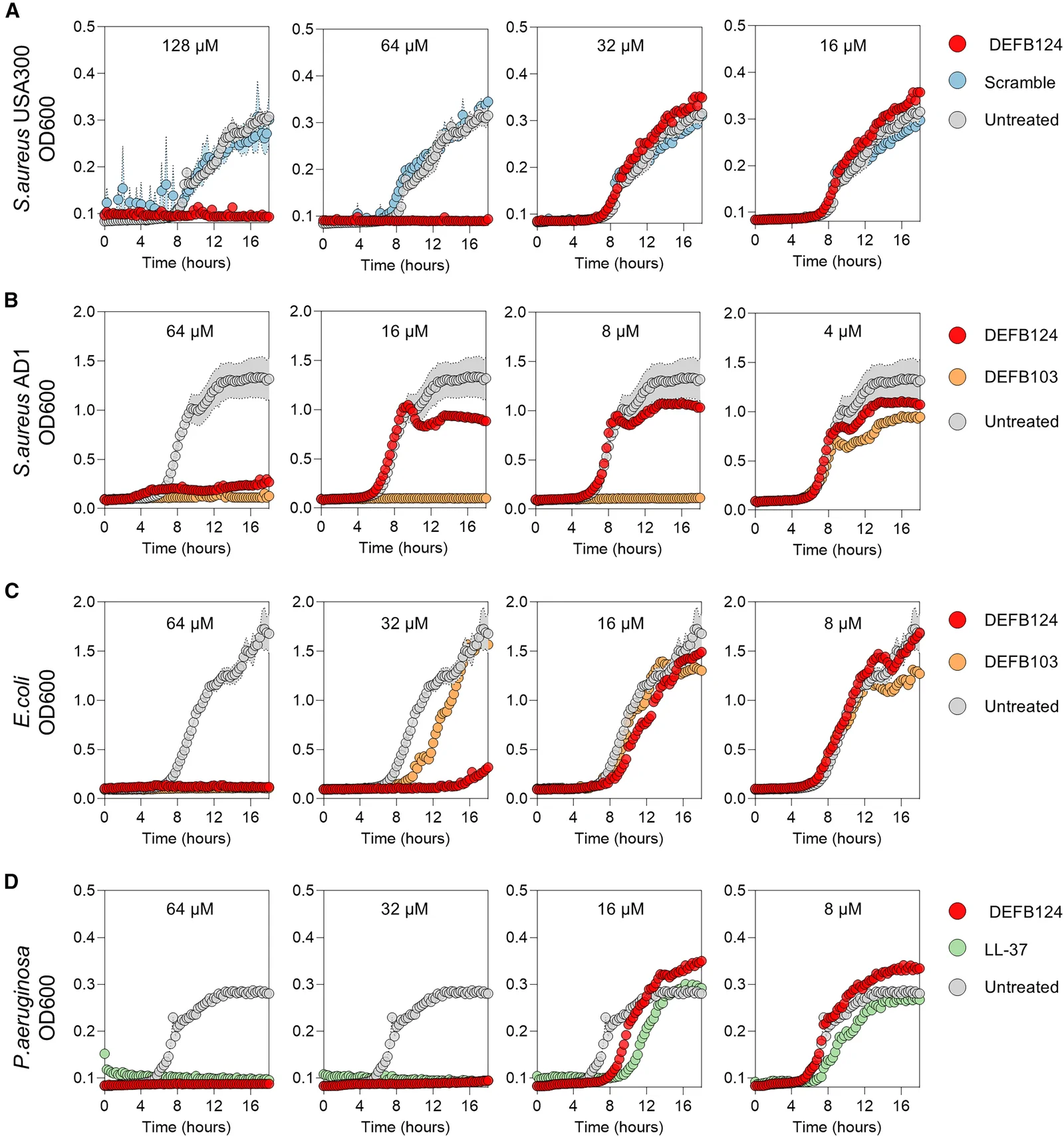
Comparative analysis of DEFB124 antimicrobial activity across bacterial species Bacterial growth was monitored by OD_600_ over time following treatment with recombinant DEFB124 at the indicated concentrations. Untreated samples and peptide controls were included where specified. (A) *S. aureus* USA300 with scrambled peptide control. (B) *S. aureus* AD1 compared with human β-defensin 3 (DEFB103). (C) *E. coli* compared with DEFB103. (D) *P. aeruginosa* compared with LL-37. See also Figure S6A.

We next used a recombinant form of the mature DEFB124 peptide expressed in yeast (*Pichia pastoris*) to assess its antimicrobial activity. Recombinant DEFB124 displayed antimicrobial activity against *S. aureus* A113 in a dose-dependent manner, with a minimum inhibitory concentration (MIC) of 64 μM (Figure 4B). Transmission electron microscopy (TEM) of *S. aureus* treated with recombinant DEFB124 revealed membrane disruption and the appearance of mesosome-like structures, indicative of membrane-targeting activity (Figure 4C). Antimicrobial activity was further confirmed against *S. aureus* USA300 (Figure 5A; MIC = 64 μM) and an AD-derived *S. aureus* clinical isolate (Figure 5B; MIC = 64 μM). Activity was also observed against the Gram-negative pathogens *Escherichia coli* (*E. coli*) (Figure 5C; MIC = 64 μM), with a significant reduction in bacterial growth at 32 μM, and *Pseudomonas aeruginosa* (*P. aeruginosa*) (Figures 5D and S6A; MIC = 32 μM). No antimicrobial activity was observed with a scrambled peptide containing the same amino acid composition as DEFB124 but an altered sequence (Figure 5A). Comparative analysis with DEFB103 showed that DEFB124 exhibits moderate activity against *S. aureus* AD1 relative to DEFB103 (MIC = 8 μM; Figure 5B), while displaying slightly higher potency against the Gram-negative bacterium *E. coli* (Figure 5C). Furthermore, comparison with cathelicidin (LL-37) revealed similar efficacy against *P. aeruginosa* (Figure 5D), although DEFB124 remained less active against *S. aureus* (data not shown).

Together, these findings demonstrate that DEFB124 exhibits sequence-specific antimicrobial activity against Gram-positive *S. aureus* and relatively enhanced activity against Gram-negative bacteria, comparable in part to established AMPs. Coupled with its canonical β-defensin structural features, including amphipathicity, cationic surface properties, and conserved cysteine architecture, these results support a functional role for DEFB124 in host defense against clinically relevant skin pathogens.

### Defb25 contributes to cutaneous antibacterial defense during *S. aureus* skin infection

**Figure 6:**
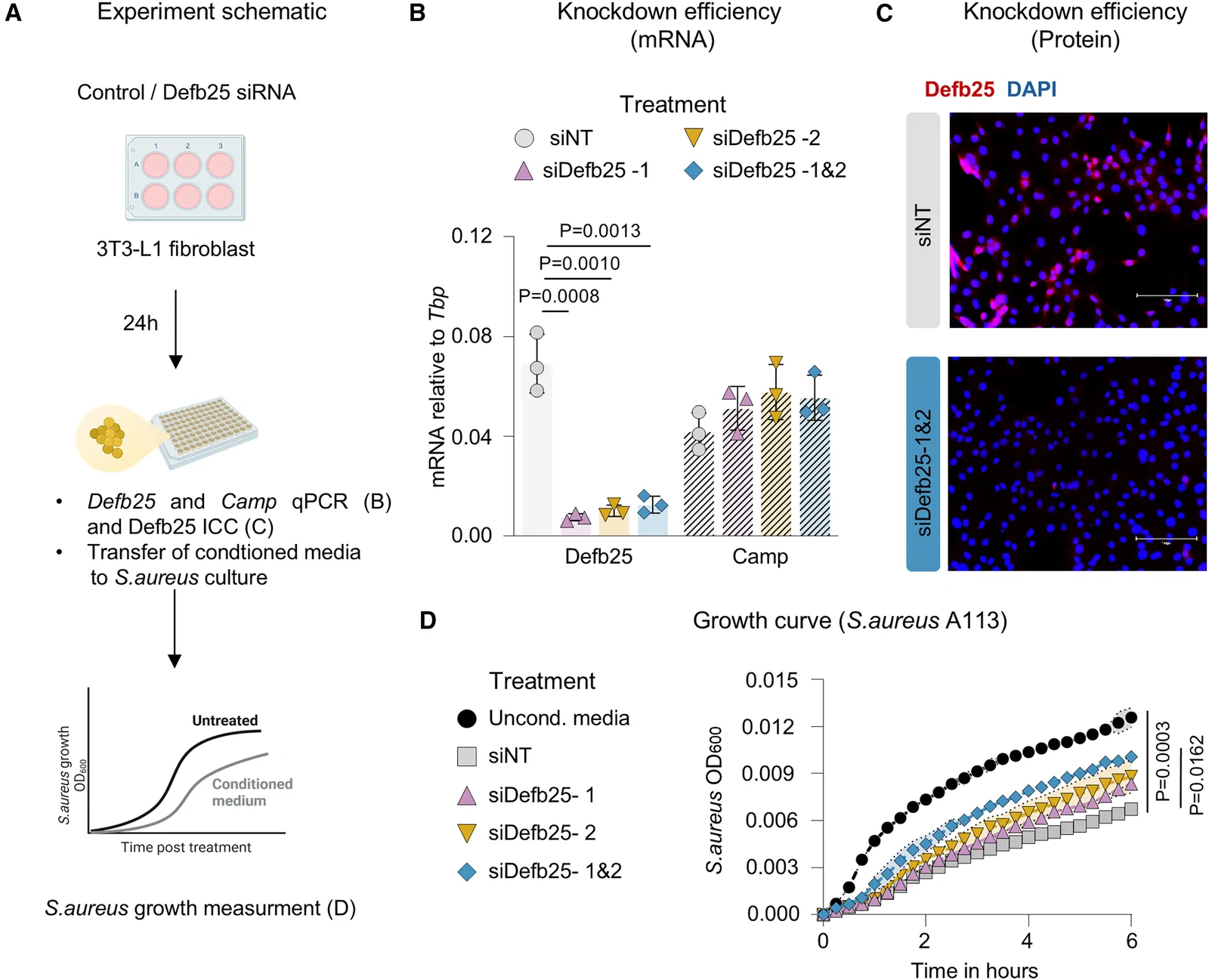
Reduced antimicrobial activity following Defb25 knockdown in 3T3-L1 cells (A) Experimental schematic. 3T3-L1 fibroblasts were transfected with non-targeting control siRNA (siNT) or Defb25-targeting siRNAs (siDefb25-1, siDefb25-2, or a combination). After 24 h, RNA and protein were collected to assess knockdown efficiency by qPCR and immunofluorescence respectively. Conditioned media from transfected fibroblasts were transferred to *S. aureus* cultures to assess antimicrobial activity. See also Figures S6B and S6C. (B) qPCR analysis of Defb25 and Camp mRNA expression following siRNA-mediated knockdown. Data are normalized to Tbp (2^∧^−ΔCt). *n* = 3 biological replicates. Statistical significance was determined by the Mann-Whitney test. (C) Representative immunofluorescence images confirming reduced Defb25 protein expression in siDefb25-treated fibroblasts compared with non-targeting control siRNA (siNT). Defb25 is shown in red, and nuclei are counterstained with DAPI (blue). Scale bars, 150 μm. (D) Growth curves of *S. aureus* A113 cultured in fibroblast-conditioned media. Conditioned media from siDefb25-transfected fibroblasts exhibited reduced antimicrobial activity, resulting in increased bacterial growth compared with siNT-conditioned media, although growth remained lower than in unconditioned media. *n* = 3. Statistical analysis was performed on OD values at 6 h using one-way ANOVA with Tukey’s multiple-comparisons test.

Having demonstrated that recombinant DEFB124 exhibits direct antimicrobial activity against *S. aureus*, we next asked whether endogenous Defb25 contributes to fibroblast-mediated antibacterial defense. 3T3-L1 fibroblasts were transfected with siRNAs targeting Defb25 (two individual siRNAs and a pooled combination) (Figure 6A). After 24 h, qPCR confirmed efficient knockdown of Defb25, while expression of cathelicidin antimicrobial peptide (Camp), an AMP from an unrelated gene family, remained unaffected (Figure 6B). Reduction at the protein level was verified by immunofluorescence staining (Figure 6C).

To determine the functional consequence of Defb25 depletion, conditioned media from siRNA-treated fibroblasts was transferred to *S. aureus* A113 cultures. Supernatants derived from control fibroblasts inhibited bacterial growth, whereas knockdown of Defb25 significantly impaired this antimicrobial effect (Figure 6D). Quantification of bacterial burden by colony-forming unit (CFU) enumeration and optical density measurements further confirmed increased bacterial growth in cultures exposed to Defb25-deficient supernatants compared to controls (Figures S6B and S6C), supporting a functional contribution of fibroblast-derived Defb25 to antibacterial activity.

**Figure 7:**
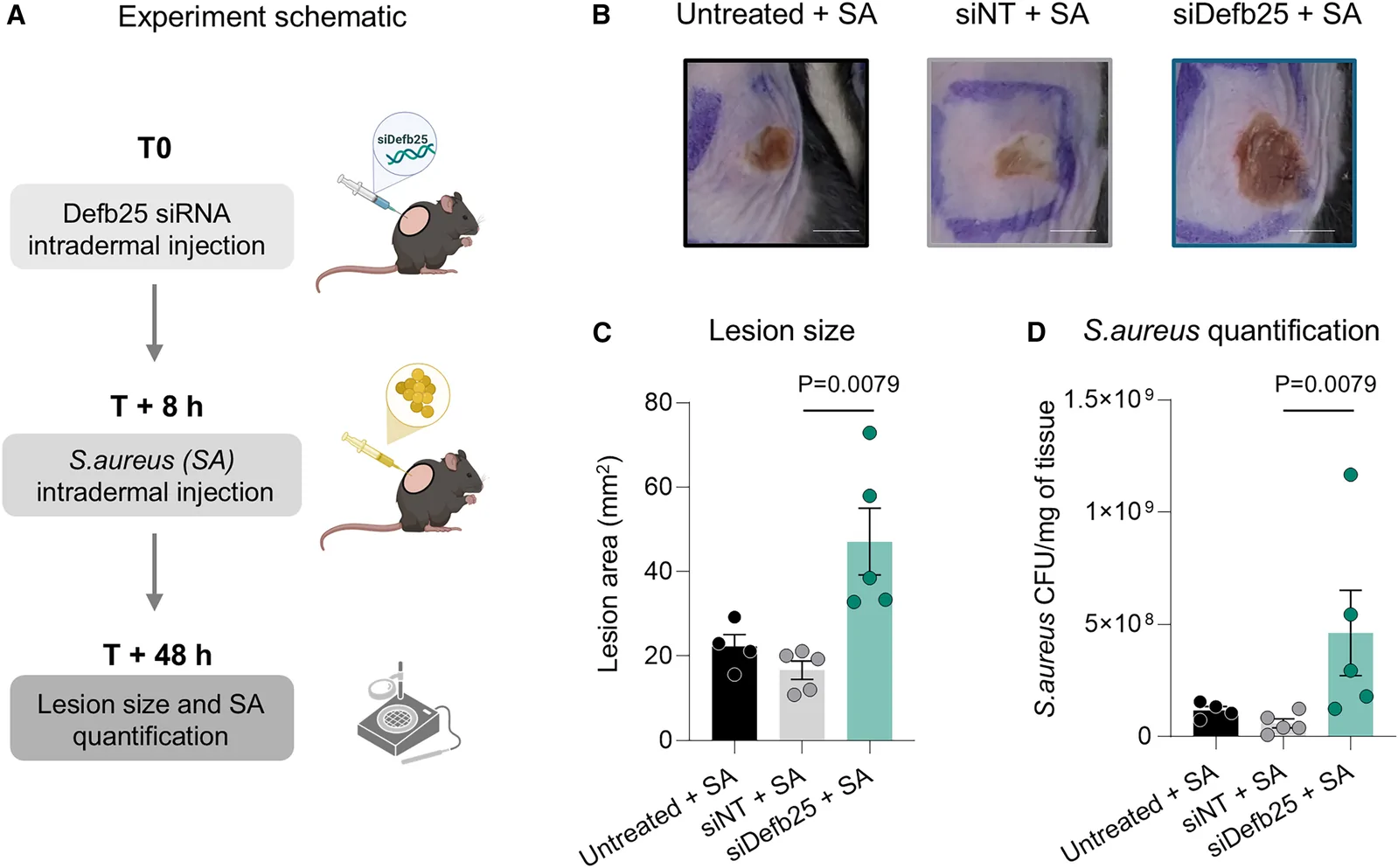
Defb25 contributes to cutaneous antibacterial defense against *S. aureus* skin infection (A) Experimental schematic of *in vivo* Defb25 knockdown and intradermal *S. aureus* infection. Mice received intradermal injection of Defb25 siRNA (siDefb25) or non-targeting control siRNA (siNT). 8 h later, mice were challenged intradermally with *S. aureus* (SA). Lesion size and bacterial burden were assessed 48 h after the initial siRNA injection. See also Figure S6H. (B) Representative images of skin lesions from untreated + SA, siNT + SA, and siDefb25 + SA groups 48 h after *S. aureus* infection. Scale bars, 50 mm. (C) Quantification of lesion area following *S. aureus* infection in the indicated groups. (D) Quantification of *S. aureus* colony-forming units (CFUs) recovered from infected skin lesions. Data are presented as mean ± SEM. Statistical significance was determined using the Mann-Whitney test. Each dot represents an individual mouse (*n* = 4–5 mice per group).

To further assess the *in vivo* contribution of endogenous Defb25 to antibacterial defense, mice were intradermally treated with Defb25-targeting siRNA prior to infecting them with *S. aureus* (Figure 7A). Defb25 knockdown elevated *S. aureus* burden and increased lesion severity in infected skin compared to negative control siRNA-treated animals, as demonstrated by changes in lesion size and CFU quantification (Figures 7B–7E). These findings support a protective role for endogenous Defb25 against cutaneous *S. aureus* infection.

Consistent with these findings, local treatment with recombinant DEFB124 reduced *S. aureus* burden and attenuated infection severity *in vivo*, further supporting the antimicrobial function of this β-defensin in the skin (Figures S6D–S6G). Together, these findings demonstrate that DEFB124 functions as an effective antimicrobial effector *in vivo*, reducing *S. aureus* burden and attenuating infection severity in the skin.

## Discussion

Comparison of resolving AD lesions with matched baseline samples revealed upregulation of DCD and DEFB124, suggesting that these peptides may contribute to the restoration of antimicrobial defense during tissue recovery.

DCD is a well-characterized anionic AMP.^33^^,^^34^^,^^35^ In contrast, the function of DEFB124 remains poorly understood. Although transcriptomic analyses and other studies have reported altered DEFB124 expression and regulation in various disease contexts and infection,^36^^,^^37^^,^^38^^,^^39^ its cellular sources and role in skin immunity have not been clearly defined.

Unlike well-characterized skin-expressed β-defensins (DEFB1, DEFB4, and DEFB103) located on chromosome 8p23.1, DEFB124 resides in a distinct cluster on chromosome 20q11.1.^29^ Defensins within this cluster are thought to display distinct tissue-restricted expression patterns and may have functions beyond canonical antimicrobial activity.^14^^,^^40^^,^^41^^,^^42^ Public datasets indicate that DEFB124 is expressed across multiple epithelial-associated tissues, raising the possibility of a broader role in barrier defense. DEFB124 corresponds to the murine Defb25, which we refer to as DEFB124/Defb25 when discussing findings across human and mouse systems.^29^

We report that DEFB124/Defb25 is predominantly expressed in dermal stromal cells, including dermal fibroblasts and adipocytes, in contrast to other skin-expressed β-defensins that are mainly produced by keratinocytes. These findings broaden the cellular sources of defensins beyond their established expression in epithelial cells and granulocytes.^43^ DEFB124/Defb25 was also detected in melanocytes, raising the possibility of additional context-dependent functions, although this will require further investigation. The current dataset did not comprehensively capture skin muscle cells, which could be explored in future studies.

Type 2 cytokines and *S. aureus* are central to AD pathogenesis, where IL-4- and IL-13-driven inflammation suppresses antimicrobial defenses and promotes *S. aureus* colonization and invasion into the skin.^22^^,^^44^ Given the role of *S. aureus* as both a driver of inflammation and a common cause of skin infection,^45^ we investigated the regulation of Defb25 in response to *S. aureus* challenge and inflammatory cues. Defb25 expression increased following *S. aureus* exposure and stimulation with type 1 and type 3 cytokines (IFN-γ and IL-17), whereas type 2 cytokines suppressed its expression, consistent with patterns observed for other β-defensins.^46^^,^^47^ Spatial transcriptomics revealed that, unlike Defb1, Defb2, and Defb14, which were largely confined to the epidermis, Defb25 was enriched in the dermal compartment during *S. aureus* infection. Together with scRNA-seq and protein-level analyses, these findings further support a predominantly dermal stromal source of DEFB124/Defb25.

The increased DEFB124 expression observed in improving lesions may reflect restoration of antimicrobial peptide production during tissue repair or relief of type 2 cytokine-mediated suppression following effective therapy, consistent with the mechanism of dupilumab, which blocks IL-4Rα signaling.^48^ Supporting this, IL-4Rα knockdown in 3T3-L1 fibroblast-like preadipocytes treated with type 2 cytokines restored Defb25 expression, indicating direct regulation by type 2 cytokine signaling. However, longitudinal sampling of matched healthy, lesional, and post-treatment skin will be required to define the temporal dynamics of DEFB124 expression and clarify its contribution to therapeutic responses in AD.

Structural analysis of DEFB124 revealed cationic and amphipathic features characteristic of membrane-disrupting AMPs, with an amphipathic profile resembling that of DEFB103, a potent AMP active against *S. aureus*.^49^^,^^50^ Consistent with these properties, recombinant DEFB124 exhibited antimicrobial activity and reduced bacterial burden and disease severity *in vivo*, while *in vivo* knockdown of Defb25 increased *S. aureus* burden and infection severity, supporting a protective role for DEFB124/Defb25 in host defense. Notably, DEFB124 exhibited modestly enhanced potency against Gram-negative bacteria compared with Gram-positive *S. aureus in vitro* when evaluated alongside established AMPs, including DEFB103 and LL-37. The distinct amino acid composition of DEFB124, including increased proline content and a comparatively less cationic profile than other β-defensins, may contribute to this differential activity, potentially by promoting structural adaptability and altered membrane interactions.^51^^,^^52^

In addition to their direct antimicrobial activity, several defensins have been reported to exert immunomodulatory functions.^8^^,^^53^ Our data show that DEFB124 treatment was associated with increased expression of immune-associated genes, including S100A8, in *S. aureus*-infected skin. Thus, DEFB124 may influence local immune responses in addition to acting as a direct antimicrobial effector.

Together, our findings establish DEFB124 as a previously unrecognized defensin expressed in the skin, thereby expanding the repertoire of defensins contributing to cutaneous host defense while highlighting a distinct cellular origin compared with conventionally described skin defensins. More broadly, these results suggest that antimicrobial defense in the skin involves coordinated contributions from both epidermal and dermal compartments. Notably, restoration of defensin expression, including DEFB124 observed across diverse therapeutic strategies including topical, oral or systemic agents, may represent a shared mechanism through which clinically effective AD therapies enhance cutaneous host defense against *S. aureus*.

### Limitations of the study

Several limitations should be considered when interpreting these findings. First, this study did not include a Defb25 knockout model, which would provide definitive genetic confirmation of its function *in vivo*. However, both *in vitro* and *in vivo* siRNA-mediated knockdown of Defb25 impaired antibacterial defense and increased *S. aureus* infection severity, supporting a functional role for Defb25 in host defense. Future genetic studies will be important to further define its physiological and cell-type-specific contributions to skin immunity.

In addition, although our analyses support dermal fibroblasts as a major stromal source of DEFB124/Defb25, we were not able to entirely define the precise fibroblast subsets expressing Defb25. scRNA-seq analyses suggested enrichment within an activated inflammatory fibroblast population; however, sparse defensin transcript detection limited robust fibroblast subclustering. Notably, its predominant expression in fibroblasts suggests a role distinct from epithelial AMPs, potentially contributing to host defense within deeper dermal compartments.

Finally, although cytokines regulating Defb25 were identified, the downstream signaling mechanisms and potential immunomodulatory functions of DEFB124/Defb25 remain to be scrutinized. Further studies will be needed to define how DEFB124 contributes to inflammatory signaling within the skin microenvironment.

## Resource availability

### Lead contact

Requests for further information and resources should be directed to and will be fulfilled by the lead contact, Vijaykumar Patra (vijaykumar.patra@gmail.com).

### Materials availability

This study did not generate new unique reagents.

### Data and code availability

• This paper analyzes existing, publicly available data, accessible at https://doi.org/10.1126/sciimmunol.ads0519, https://doi.org/10.1016/j.celrep.2023.112494, https://doi.org/10.21203/rs.3.rs-7559111/v1, and https://doi.org/10.1016/j.jaci.2020.01.042.
• This paper does not report original code.
• Any additional information required to reanalyze the data reported in this paper is available from the lead contact upon request.

## Acknowledgments

The authors thank Teruaki Nakatsuji, Kellen Cavagnero, Samia Almoughrabie, and Jared Simmons for their valuable contributions to this work. We also acknowledge the University of California, San Diego Cellular and Molecular Medicine Electron Microscopy Core (UCSD-CMM-EM Core, RRID: SCR_022039) for providing access to equipment and technical support. We further thank the Bioimaging Core Facility for technical assistance as well as the Center for Medical Research (ZMF) and the Medical University of Graz for providing research infrastructure and technical facilities. This study was supported by a collaborative international project jointly funded by the 10.13039/501100002428Austrian Science Fund (FWF, Project I 4939-B) and the 10.13039/501100001665French National Research Agency (ANR, ANR-20-CE91-0002), awarded to P.W., J.-F.N., M.V., and V.P. R.L.G. was supported by grants from the 10.13039/100000002National Institutes of Health (NIH-37AI1052453, NIH-U01AI152038, and NIH-P50AR080594). V.P. also received additional funding through the Sanofi–Regeneron Pharmaceuticals Type 2 Innovation Grant and a research grant from the City of Graz. Doctoral candidate A.J. was funded by Molecular Medicine PhD Program of Medical University of Graz. His research stay at the University of California, San Diego (USA) was further supported by fellowships, including the Austrian Marshall Plan Foundation Scholarship, the Marietta Blau Fellowship from OeAD (Österreichische Austauschdienst-Gesellschaft), and the Josef Kyrle travel fund from Ö10.13039/501100006526GDV (Austrian Society of Dermatology and Venereology). The funders had no role in the study design, data collection, analysis, interpretation of the results, or the decision to publish the findings.

## Author contributions

Conceptualization, A.J., V.P., and R.L.G.; data curation, A.J., T.N., and A.R.M.; formal analysis, A.J., T.N., T.D., and H.C.; funding acquisition, P.W., M.V., V.P., R.L.G., and J.-F.N.; investigation, A.J., T.N., H.C., A.R.M., T.D., and A.S.; methodology, A.J., V.P., and R.L.G.; project administration, R.L.G., V.P., and P.W.; resources, P.W., M.V., R.L.G., and P.K.; software, A.J.; supervision, P.W., R.L.G., and V.P.; validation, R.L.G., V.P., and P.W.; visualization, A.J. and V.P.; writing – original draft, A.J.; writing – review and editing, R.L.G., V.P., P.W., A.J., M.V., and J.-F.N. All authors have critically revised the manuscript.

## Declaration of interests

R.L.G. is a co-founder, scientific advisor, consultant, and equity holder of MatriSys Biosciences.

## Declaration of generative AI and AI-assisted technologies in the writing process

The authors acknowledge the use of Academic AI (Med Uni Graz, powered by OpenAI models via ACONet) for language refinement and clarity improvements. All AI-assisted outputs were carefully reviewed and verified by the authors.

## STAR★Methods

### Key resources table

**Table:** 

REAGENT or RESOURCE	SOURCE	IDENTIFIER
**Antibodies**		

DEFB124 Polyclonal Antibody	Thermo Fisher Scientific	Cat# PA5-62302; RRID:AB_2640499
CD140a (PDGFRA) Monoclonal Antibody (APA5), eBioscience™	Thermo Fisher Scientific	Cat# 14-1401-82; RRID:AB_467491
Vimentin polyclonal antibody	Thermo Fisher Scientific	Cat# PA5-142829; RRID:AB_2933472
MART-1/Melan-A/MLANA (Melanoma Marker) Monoclonal Antibody (M2-7C10)	Thermo Fisher Scientific	Cat# 2315-MSM1-P0; RRID:AB_142033
Mouse/Rat FABP4/A-FABP Antibody	R and D Systems	Cat# AF1443; RRID:AB_2102444
Adiponectin Monoclonal Antibody (19F1)	Thermo Fisher Scientific	Cat# MA1-054; RRID:AB_557516
Goat anti-Mouse IgG (H + L) Cross-Adsorbed Secondary Antibody, Alexa Fluor™ 488	Thermo Fisher Scientific	Cat #A-11001; RRID:AB_2534069
Donkey anti-Rabbit IgG (H + L) Highly Cross-Adsorbed Secondary Antibody, Alexa Fluor™ Plus 594	Thermo Fisher Scientific	Cat #A32754; RRID:AB_2762827
Donkey anti-Goat IgG (H + L) Secondary Antibody, FITC	Thermo Fisher Scientific	Cat#16000; RRID:AB_2534674

**Bacterial and virus strains**		

*Staphylococcus aureus* strain A113	ATCC	Cat# ATCC35556
*Staphylococcus aureus* strain AD1	Joshi, Cornu et al. 2026^54^	N/A
*Pseudomonas aeruginosa* PAO1	Stover, Pham et al. 2000^55^	N/A
*Escherichia coli*	DSMZ	Cat # DSM 613/ATCC 11303
*Staphylococcus aureus* USA300	ATCC	Cat# BAA-1717

**Chemicals, peptides, and recombinant proteins**		

Recombinant human β-defensin 124	Cusabio Biotech Co., Ltd.	Cat# CSB-YP836731HU Lot# DA05796a6g0 and DA5806a6g0
LL-37	Genemed Synthesis Inc., San Antonio, Texas, USA	Custom pepitde
Scramble (sequence: LKGCYSMEFHD PWKAAYEHKLEKYSTRPEVGLCQPPP AYQLQCCRCTCT)	Peptide 2.0, Chantilly, VA, USA	Custom peptide
Recombinant Human BD-3 (β-defensin 103)	PeproTech	Cat # 300-52-250UG Lot# 0710210 C1918
Murine IL-4	PeproTech & R&D Systems	Cat#214-14-20UG and Cat# 404-ML for IL-4 and
Murine IL-13	PeproTech & R&D Systems	Cat#210-13-10UG and Cat#413-ML
Vancomycin Hydrochloride, 250 mg,	Carl Roth GmbH	Cat#0242.1
Murine IL-22	PeproTech	Cat# 210-22-50UG
Murine Il17a	PeproTech	Cat#210-17-25UG
Murine IFN-ϒ	PeproTech	Cat#315-05-100UG
Pierce™ Protease Inhibitor Tablets, EDTA-free	Thermo Fisher Scientific	Cat #A32965
Antibody Diluent	Agilent Dako	Cat #S3022
Protein Block, Serum-Free	Agilent Dako	Cat #X0909

**Critical commercial assays**		

Defb25 ELISA kit	Abexxa Ltd, Cambridge, UK	Cat# abx510212
Defb14 ELISA kit	Abexxa Ltd, Cambridge, UK	Cat# abx254765

**Deposited data**		

Human skin single cell RNA-seq data	He et al.^56^	GSE147424(https://www.ncbi.nlm.nih.gov/geo/)
Mouse skin single cell RNA-seq data	Nakatsuji et al.^23^	DDBJ sequence read archive (www.ddbj.nig.ac.jp), accession number: DRA015287
Mouse skin spatial RNA-seq data	Dokoshi et al.^57^	Available upon request
Visium CytAssist, Mouse Embryo, 11 mm Capture Area (FFPE)	10X genomics	https://www.10xgenomics.com/datasets
Mouse skin single cell RNA-seq data	Chan et al.^58^	GSE270712 (https://www.ncbi.nlm.nih.gov/geo/I)

**Experimental models: Cell lines**		

3T3-L1	ATCC	Cat# CL-173; RRID:CVCL_0123
Human fibroblast like preadipocytes	Cell Applications	Cat#802 s-05a

**Experimental models: Organisms/strains**		

C57BL/6J (C57 Black 6) mice	The Jackson Laboratory	RRID:IMSR_JAX:000664

**Oligonucleotides**		

Defb25 siRNA −1	Invitrogen	Part number AM16708, ID #506022 Lot #ASO2MUIW
Defb25 siRNA −2	Invitrogen	Part number AM16708, ID #506023 Lot #ASO2MZAE
Negative control siRNA	Invitrogen	#AM4613
Il4ra siRNA	Invitrogen	Cat# s68281

**Software and algorithms**		

SOFTWARE OR ALGORITHM	SOURCE	IDENTIFIER
Jalview	Waterhouse, Procter et al. 2009^59^	https://www.jalview.org
ChimeraX	Meng, Goddard et al. 2023,UCSF^32^	Version 1.9
BioRender	BioRender	https://biorender.com
R	R Foundation for Statistical Computing	https://www.r-project.org
Loupe Browser	10x Genomics	https://www.10xgenomics.com
Genevestigator	Nebion AG	https://genevestigator.com
GraphPad Prism	GraphPad Software	v9.5.1 https://www.graphpad.com

**Other**		

Lipofectamine™ RNAiMAX Transfection Reagent	Thermo Fisher Scientific	Cat#13778075
METAFECTENE® SI+	Biontex	Cat #T100 Lot no. ADSiP307/RK102218
MagNA Lyser Green Beads	Roche	Cat#03358941001
Fluorescence Mounting Medium	Agilent Dako	Cat#S302380-2
Target Retrieval Solution, Citrate pH 6	Agilent Dako	Cat #S2369

### Experimental model and study participant details

#### Mouse experiments

Female wild-type C57BL/6J mice (6–8 weeks of age) were used for all *in vivo* experiments. Mice were maintained under specific pathogen-free (SPF) conditions in a temperature- and humidity-controlled facility on a 12-h light/dark cycle with food and water provided *ad libitum*. Animal experiments performed at the University of California San Diego were approved by the Institutional Animal Care and Use Committee (IACUC) of the University of California San Diego (Protocol No. S09074). Additional Animal experiments were conducted at the Medical University of Graz were approved by the Austrian Federal Ministry of Education, Science and Research (Approval No. GZ 2022-0.673.036). Only female mice were used in this study, and the potential influence of sex on the findings should be considered when interpreting the results.

#### Human primary cell cultures

Primary human keratinocytes, dermal fibroblasts, and subcutaneous preadipocytes were isolated from human skin explants obtained with approval from the Ethics Committee of the Medical University of Graz, Austria (Approval No. EK: 28-151 ex 15/16). Human tissues were collected and processed in accordance with institutional ethical guidelines and applicable regulations. Informed consent was obtained from all subjects. Primary human preadipocytes were isolated from 4 female donors (age range 31–39 years; Table S6). Primary human dermal fibroblasts and keratinocytes were isolated from 3 donors (2 female, 1 male; age range 31–42 years; Table S7). Subcutaneous preadipocytes were differentiated into mature adipocytes *in vitro* over a 10-day differentiation protocol, with samples collected at days 4, 8, and 10 (D4, D8, D10) from the same donor cohort described above. Full donor characteristics, including collection date and BMI, are provided in Tables S6 and S7. Donor ancestry, race, and ethnicity were not recorded. Cells from each donor were processed and analyzed independently, with each donor’s cells serving as an independent biological replicate.

#### Cell lines

The 3T3-L1 murine cell line and human fibroblast-like preadipocytes were used as described in the relevant method details. Sources, catalog numbers, and identifiers are provided in the key resources table. Cell lines were not authenticated via short tandem repeat (STR) profiling. No additional mycoplasma testing was performed in-house; mycoplasma testing was performed by ATCC as part of their standard quality control prior to shipment.

#### Microbial strains

*S. aureus strains* AD1, USA300, and A113, Pseudomonas aeruginosa PAO1, and Escherichia coli were used in this study. Sources and identifiers are provided in the key resources table.

### Method details

#### Immunofluorescence staining of human skin sections

Paraffin-embedded human skin tissues were sectioned at 7 μm thickness. Sections were deparaffinized in xylene and rehydrated through a graded ethanol series (50–90%), followed by rinsing in PBS. Antigen retrieval was performed in Dako Target Retrieval Solution Citrate Buffer pH 6 (1:10 dilution) for 30 min, followed by a 20-min cool-down. Sections were washed in PBS and blocked for 1 h at room temperature using Dako blocking solution. Primary antibodies were diluted in Dako Antibody Diluent and incubated overnight at 4°C. Antibodies included anti-DEFB124 (Dilution 1:200), together with antibodies against PDGFRα (Dilution 1:100), vimentin (1:100), MART-1/Melan-A (1:100), and FABP4 (1:100) for co-staining of specific skin cell populations (see key resources table). After washing in PBS, sections were incubated with fluorescent secondary antibodies for 60 min at room temperature. Nuclei were counterstained with DAPI, and slides were mounted using Dako Fluorescence Mounting Medium. Images were acquired using a Nikon A1 Confocal Microscope.

#### Hydrophobicity analysis and structural visualization

The peptide sequences of human defensins DEFB124, DEFB103A, DEFB4A, and DEFB1 were retrieved from the UniProt database (https://www.uniprot.org) using accession numbers Q8NES8, P81534, O15263, and P60022, respectively. To analyze comparative biochemical properties, including the hydrophobic ratio, GRAVY (Grand Average of Hydropathy) score, and net charge, the Antimicrobial Peptide Database (APD3; Center for Computational Biology, University of Nebraska, Lincoln, NE, USA) was used.^60^ For structural analysis, three-dimensional structures of the defensins were downloaded from the AlphaFold Protein Structure Database (DeepMind, London, UK; EMBL-EBI, Hinxton, UK) via UniProt. These structures were visualized using UCSF ChimeraX (version 1.9]^33^; UCSF Resource for Biocomputing, Visualization, and Informatics, San Francisco, CA, USA), which has built-in tools for mapping hydrophobic, hydrophilic, and charged residues on protein surfaces.

#### Antimicrobial assay using recombinant DEFB124

Recombinant human β-defensin 124 (DEFB124) used for experiments was produced in a yeast, *Pichia pastoris* X33. Upon receipt, the lyophilized peptide was reconstituted in sterile double-distilled water (ddH_2_O), aliquoted, and the solvent was subsequently removed using a SpeedVac SRF110 refrigerated centrifugal vacuum concentrator (Thermo Fisher Scientific, Waltham, MA, USA). The resulting aliquots were stored at −80°C until further use.

Antimicrobial activity of antimicrobial peptides was assessed as previously described.^23^ Briefly, recombinant DEFB124, DEFB103, LL-37, or a scrambled control peptide (sequence: LKGCYSMEFHDPWKAAYEHKLEKYSTRPEVGLCQPPPAYQLQCCRCTCT) was reconstituted and diluted in 90% RPMI and 10% tryptic soy broth (TSB) to the desired working concentration. Bacterial cells were washed with PBS and resuspended in 90% RPMI/10% TSB mixture. Bacteria (1 × 10^5^ CFU/mL) were then incubated with or without DEFB124, LL-37, or the scrambled control peptide in 100 μL total volume per well in a 96-well plate at 37°C for 24 h. In some experiments, bacterial growth kinetics were assessed by measuring optical density at 600 nm (OD_600_) over an 18–24-h period using a microplate reader (SpectraMax, Molecular Devices). For colony-forming unit (CFU) quantification, bacterial cultures were serially diluted and plated using the easySpiral automatic plater in either exponential or circular mode (Interscience, Saint-Nom-la-Bretèche, France) or plated manually for selected experiments. After 24 h of incubation, CFUs were counted using automated colony counting software provided by Interscience. In selected assays, qualitative differences in turbidity among treatment groups were visually documented using a Google Pixel 6 or Pixel 9 smartphone camera.

#### Transmission electron microscopy

*S. aureus A113* was incubated in 90% RPMI media and 10% TSB containing DEFB124 (128 μM), at 37°C for 2 h. Bacterial cells were washed with PBS twice and immediately fixed by resuspending in modified Karnovsky’s fixative for 4 h, followed by 1% osmium tetroxide in 0.1 M cacodylate buffer for 1 h on ice. The cells were stained with 2% uranyl acetate for 1 h on ice, dehydrated in a graded series of ethanol (50–100%), washed with acetone, and then embedded with Durcupan. Sections (60 nm) were post-stained with 2% uranyl acetate for 5 min and Sato’s lead stain for 1 min. Grids were viewed using a JEOL JEM-1400Plus (JEOL, Peabody, MA) transmission electron microscope and photographed using a Gatan OneView 4 K digital camera (Gatan, Pleasanton, CA).

#### Antimicrobial peptide screening

Skin biopsy transcriptomic datasets from clinical studies of atopic dermatitis (AD) patients treated with dupilumab,^26^ crisaborole,^24^ gusacitinib,^25^ fezakinumab,^27^ or ciclosporin^26^ were obtained from previously published studies. Genes encoding antimicrobial peptides and proteins, including defensins, cathelicidin, members of the S100A family, and RNases, were screened for changes in expression across these datasets.

Fold-change values were extracted from the processed datasets accompanying the original publications when available. In several microarray studies (fezakinumab, ASN002, and crisaborole), expression changes were reported as signed fold-change values (fold regulation), in which positive values indicate increased expression and negative values indicate decreased expression relative to baseline.

For datasets in which fold-change values were not directly provided (dupilumab and ciclosporin), fold changes were calculated by dividing the mean expression values reported for treated samples by the corresponding mean baseline expression values in the deposited datasets, specifically comparing responders at 3 months post-treatment with baseline lesional samples. In these cases, fold-change values were expressed as expression ratios, where values greater than 1 indicate increased expression and values less than 1 indicate decreased expression relative to baseline.

Because only aggregated expression values were available and individual responder-level data were not accessible, statistical testing could not be performed for the dupilumab and ciclosporin datasets. Sample timepoints and corresponding expression values of shortlisted AMPs are summarized in Table S1.

#### siRNA-mediated knockdown of Defb25 and antimicrobial activity evaluation

To investigate the role of Defb25 in antimicrobial activity, confluent 3T3-L1 fibroblasts were transfected with either negative control siRNA or 30 nM Defb25-targeting siRNA-1 and siRNA-2, both supplied under part number AM16708 (Thermo Fisher Scientific, Waltham, MA, USA). Transfections were carried out using Lipofectamine RNAiMAX Transfection Reagent (Thermo Fisher Scientific, Waltham, MA, USA) according to the manufacturer’s instructions in antibiotic-free DMEM medium supplemented with 10% fetal bovine serum (FBS) and 1% GlutaMAX. After 24 h of incubation, total RNA was extracted from the cells and subjected to quantitative PCR to assess expression levels of Defb25 and Camp. In parallel, 100 μL of conditioned media from each condition was collected and transferred to 100 μL of a *S. aureus* A113 suspension (1 × 10^5^ CFU/mL) in 90% DMEM and 10% TSB in a 96-well plate. Bacterial growth was measured for 6 h by monitoring optical density at 600 nm (OD_600_) using a microplate reader.

#### Immunocytochemistry

Twenty-four hours post-transfection, 3T3-L1 cells were rinsed twice with PBS containing calcium and magnesium, then fixed in 4% paraformaldehyde for 10 min at room temperature. After three PBS washes, cells were permeabilized using 0.1% Triton X-100 in D-PBS for 5 min. Blocking was performed with Dako blocking buffer for 1 h at room temperature, followed by incubation with Rabbit anti-DEFB124 (dilution 1:200) for 2 h at room temperature. After three PBS washes, cells were incubated with Alexa Fluor 594 Donkey anti-Rabbit IgG, 1:500 diluted in Dako diluent for 1 h at room temperature. Following final PBS washes, plastic chamber walls were removed as per the manufacturer’s instructions (Nunc Lab-Tek II Chamber Slide System, Thermo Fisher Scientific, Waltham, MA, USA), and slides were mounted using ProLong Gold Antifade Mountant with DAPI.

#### DEFB124/Defb25 expression analysis in skin using single-cell RNA-Seq data

Publicly available scRNA-seq datasets of human and mouse skin were used to determine the cellular distribution of DEFB124 and Defb25. For murine and human skin scRNAseq datasets, preprocessed files (.rds) with clustering as reported^23^^,^^58^^,^^61^ were used. Expression of DEFB124/Defb25 was evaluated across annotated cell populations and visualized using UMAP to identify predominant expressing cell types. All analyses were performed in R using packages including Seurat and SingleR. Custom scripts used for data processing and visualization are available upon request.

#### Effect of type 2 cytokines with or without siRNA-mediated knockdown of Il4Ra

Mouse fibroblasts, including 3T3-L1 and primary mouse dermal fibroblasts, were cultured in DMEM supplemented with 10% FBS, 1% GlutaMAX, and 1% penicillin-streptomycin unless otherwise noted. For siRNA knockdown experiments targeting the Il4ra gene, 4.0 × 10^4^ 3T3 cells were seeded in 0.4 mL of complete DMEM in 24-well plates and incubated at 37°C in a 5% CO_2_ humidified incubator (Day −3). On Day 0, upon reaching ∼80–90% confluency, the medium was replaced with 250 μL of fresh DMEM containing 10% FBS and 1% GlutaMAX (excluding penicillin-streptomycin). siRNA transfection was performed by preparing a mixture of siRNA (final concentration 30 nM) and lipofection reagent in Opti-MEM (solutions A and B), which was added as 250 μL per well (Thermo Fisher Scientific; Silencer Negative Control #1, Cat# AM4611; Il4ra siRNA, Cat# s68281). After 24 h (Day 1), the medium was replaced with 400 μL DMEM containing 1% penicillin-streptomycin and 1% GlutaMAX. Cells were stimulated with recombinant cytokines for 24 h: IL-4 (50 ng/mL), and IL-13 (50 ng/mL), all obtained from R&D Systems (Cat# 404-ML for IL-4 and 413-ML for IL-13). The next day the cells were harvested using 0.25% trypsin-EDTA, and total RNA was extracted using standard protocols. cDNA was synthesized, and Defb25 gene expression analysis was performed via qPCR.

#### *S. aureus* infection model and cytokine stimulation

Skin infection experiments were performed following established protocols.^43^ Briefly, 6–8-week-old C57BL/6J female mice had their dorsal hair shaved and chemically depilated using Nair cream. Mice were then intradermally injected with 50 μL of *S. aureus* (5 × 10^6^ CFU) prepared from mid-logarithmic phase cultures in phosphate-buffered saline (PBS). The *S. aureus* strains USA300 and AD1 were used for these experiments. Skin tissues (6–8 mm) adjacent to the abscess center were collected at 6-, 48-, and 72-h post-infection for downstream analyses. Tissue samples were homogenized in 1 mL PureLink RNA lysis buffer (12183025, Thermo Fisher Scientific, Waltham, MA, USA) for RNA extraction. Complementary DNA (cDNA) was synthesized from the isolated RNA, and Defb25 gene expression was quantified by qPCR.

For cytokine stimulation experiments, recombinant murine IL-22, IL-17A, IFN-γ, and a mixture of IL-4 and IL-13 (1 μg/mL each) were reconstituted in PBS and injected intradermally into the dorsal skin of mice. The dorsal skin had been shaved and chemically depilated with Nair cream one day prior, as described for the infection model. Skin samples were harvested 6 h after cytokine injection. RNA was isolated, and qPCR was performed to assess Defb25 expression and marker genes associated with inflammatory activation.

#### Spatial transcriptomics data analysis

Spatial transcriptomics data of a healthy FFPE mouse embryo were downloaded from the 10x Genomics public dataset repository (file: CytAssist_11 mm_FFPE_Mouse_Embryo). Visualization of Defb25 expression (log2 scale) was performed directly using Loupe Browser (v6.5.1, 10x Genomics, Pleasanton, CA, USA), without any downstream modification or re-analysis.

To analyze defensin expression following *S. aureus* infection, spatial transcriptomics data from mouse skin two days after intradermal injection with *S. aureus* (USA300 strain) or PBS (Vehicle) were utilized. These data are included in the study by Dokoshi et al.^57^ Processed data were provided as a Seurat object (.rds), with upstream processing, including SCTransform() normalization, PCA and UMAP dimensionality reduction, clustering using FindNeighbors() and FindClusters(), and marker gene identification via FindAllMarkers(), already completed. The Seurat object was imported into R (v2024.04.2), and Defb1, Defb14, Defb2, and Defb25 gene expression were visualized using standard Seurat plotting functions.

#### *In vivo* intradermal *S. aureus* infection and DEFB124 peptide treatment

Mouse skin was infected intradermally with 50 μL of *S. aureus* (5 × 10^6^ CFU). To evaluate the *in vivo* antimicrobial activity of DEFB124, 50 μL of vehicle control, 128 μM DEFB124 peptide, or 50 μM vancomycin was administered intradermally at the same site 1 h after bacterial inoculation. Twenty-four hours after treatment, Infection severity was evaluated using a semi-quantitative scoring system based on erythema (0–3), swelling (0–3), and ulceration (0 or 2). Then dorsal skin tissues were collected and transferred into MagNA Lyser tubes prefilled with ceramic beads (Cat# 03358941001, Roche) containing 1 mL sterile PBS. Tissue homogenization was performed using a MagNA Lyser at 6000 rpm for 30 s. Homogenates were serially diluted and plated on tryptic soy agar (TSA) and/or chromID *S. aureus* Elite agar (bioMérieux). Plates were incubated overnight at 37°C, and bacterial burden was quantified as colony-forming units (CFU) per gram of tissue using ImageJ.

#### *In vivo* intradermal *S. aureus* infection and siRNA-mediated Defb25 knockdown

For *in vivo* knockdown experiments, negative control siRNA or Defb25 siRNA was mixed with Metafectene and 1× buffer according to the manufacturer’s instructions. A total volume of 50 μL containing 60 pmol siRNA was injected intradermally into the skin. Eight hours after siRNA administration, following resolution of the injection depot, 50 μL of *S. aureus* was injected intradermally at the same site. Forty-eight hours after the initial siRNA injection, clinical images were acquired and tissue samples were harvested for bacterial colony enumeration as described above. Lesion areas were quantified using ImageJ.

#### Human preadipocyte isolation and culture

Subcutaneous preadipocytes were isolated from human skin explants obtained with approval from the Ethics Committee of the Medical University of Graz, Austria (EK: 28–151 ex 15/16). Briefly, approximately 100 g of subcutaneous adipose tissue was dissected from skin explants, cut into ∼1 cm pieces, washed with 1× PBS, and cleared of connective tissue and blood vessels. The adipose tissue was finely minced and enzymatically digested in PBS containing 0.2% (w/v) collagenase II and 0.5% (w/v) bovine serum albumin (BSA) for 1.5 h at 37°C with regular inversion. Following digestion, the suspension was filtered through sterile gauze and centrifuged at 300 × g for 5 min to isolate the stromal vascular fraction (SVF). The SVF-containing pellet was collected, washed with PBS, sequentially filtered through 70 μm and 40 μm cell strainers, and centrifuged between filtration steps. Residual erythrocytes were removed by incubation with RBC lysis buffer for 10 min at room temperature. Cells were subsequently washed, counted using a Neubauer chamber, and seeded at a density of 50,000 cells/cm^2^ in standard culture-treated flasks containing cultivation medium under standard culture conditions (37°C, 5% CO_2_). Preadipocytes were maintained at 37°C and 5% CO_2_ and cultured to 70–80% confluency. The composition of the cultivation media is shown in Table S3.

#### Adipogenic differentiation

For adipogenic differentiation, preadipocytes were seeded at a density of 10,000 cells/cm^2^ and cultured to confluency. Cell differentiation was induced using adipogenic induction medium (Table S4), which was replaced every 48 h. After 4 days of induction, cells displayed multilocular lipid droplets and were switched to adipocyte growth medium (Table S5). Medium changes were subsequently performed carefully due to the increased fragility and detachment tendency of mature adipocytes.

#### Western blot

Proteins were separated using Novex 10–20% Tricine SDS-PAGE gels (#EC66255BOX, Thermo Fisher Scientific, Waltham, MA, USA) and transferred onto PVDF membranes (#1620174, Bio-Rad Laboratories, Hercules, CA, USA) using the Trans-Blot transfer system. Membranes were blocked in 5% BSA in TBST at room temperature with gentle shaking, followed by overnight incubation at 4°C with DEFB124 Polyclonal Antibody at a 1:200 dilution in 5% BSA-TBST. After washing, membranes were incubated with an IRDye-conjugated secondary antibody (1:2000 in 3% BSA-TBST) at room temperature. Signal detection was performed using the Odyssey Fluorescent Imaging System (LI-COR Biosciences, Lincoln, NE, USA), and bands were visualized using Image Studio software.

#### Defb14 and Defb25 ELISA

Adjacent skin biopsies were collected at 6 h and 72 h after *S. aureus* infection. Tissue samples were lysed in 200 μL of T-PER Tissue Protein Extraction Reagent supplemented with Protease Inhibitor Cocktail. Samples were placed in MagNA Lyser tubes prefilled with ceramic beads and homogenized using a MagNA Lyser at 6500 rpm for 30 s, repeated three times. Lysates were then centrifuged at maximum speed for 5 min, and the supernatants were collected for further analysis. Total protein concentration of the lysates was determined using a BCA Protein Assay according to the manufacturer’s instructions. Levels of Defb14 and Defb25 were measured using ELISA kits from Abeexa following the manufacturer’s protocols. Absorbance was measured at 450 nm using a microplate reader, and defensin concentrations were calculated based on the standard curves obtained from standards provided with the kits. To account for differences in tissue input and extraction efficiency, defensin levels were normalized to the total protein concentration of each lysate as determined by the BCA assay. Results were expressed as defensin levels relative to total protein.

### Quantification and statistical analysis

Statistical analyses were performed using GraphPad Prism v9.5.1 (GraphPad Software, San Diego, CA, USA; https://www.graphpad.com). Data distribution was assessed using the Shapiro–Wilk normality test with a significance threshold of α = 0.05. For normally distributed datasets, one-way ANOVA followed by Tukey’s multiple comparison test was used to evaluate differences among groups. For data that did not meet normality assumptions, the Kruskal–Wallis test with Dunn’s post hoc correction was applied. Comparisons between two independent groups were analyzed using the Mann–Whitney U test. For experiments involving the same mice injected at different sites, paired *t*-tests were used following assessment of normality. Data are presented as mean ± SEM, unless otherwise specified.
