# Supplementary material for: A stromal β-defensin, DEFB124, contributes to cutaneous host defense against Staphylococcus aureus
Source: iScience. 2026 Aug 27;29(9):117306. doi: 10.1016/j.isci.2026.117306 (PMC13546879; doi:10.1016/j.isci.2026.117306)
Supplement: Supplementary file 1 [file mmc1.pdf]

iScience, Volume *29*

Supplemental information

A stromal β-defensin, DEFB124, contributes to cutaneous host defense against *Staphylococcus aureus*

Aaroh Joshi, Tomofumi Numata, Andrea Roso Mares, Hung Chan, Tatsuya Dokoshi, Anna Schwarz, Petra Kotzbeck, Jean-François Nicolas, Marc Vocanson, Peter Wolf, Vijaykumar Patra, and Richard L. Gallo

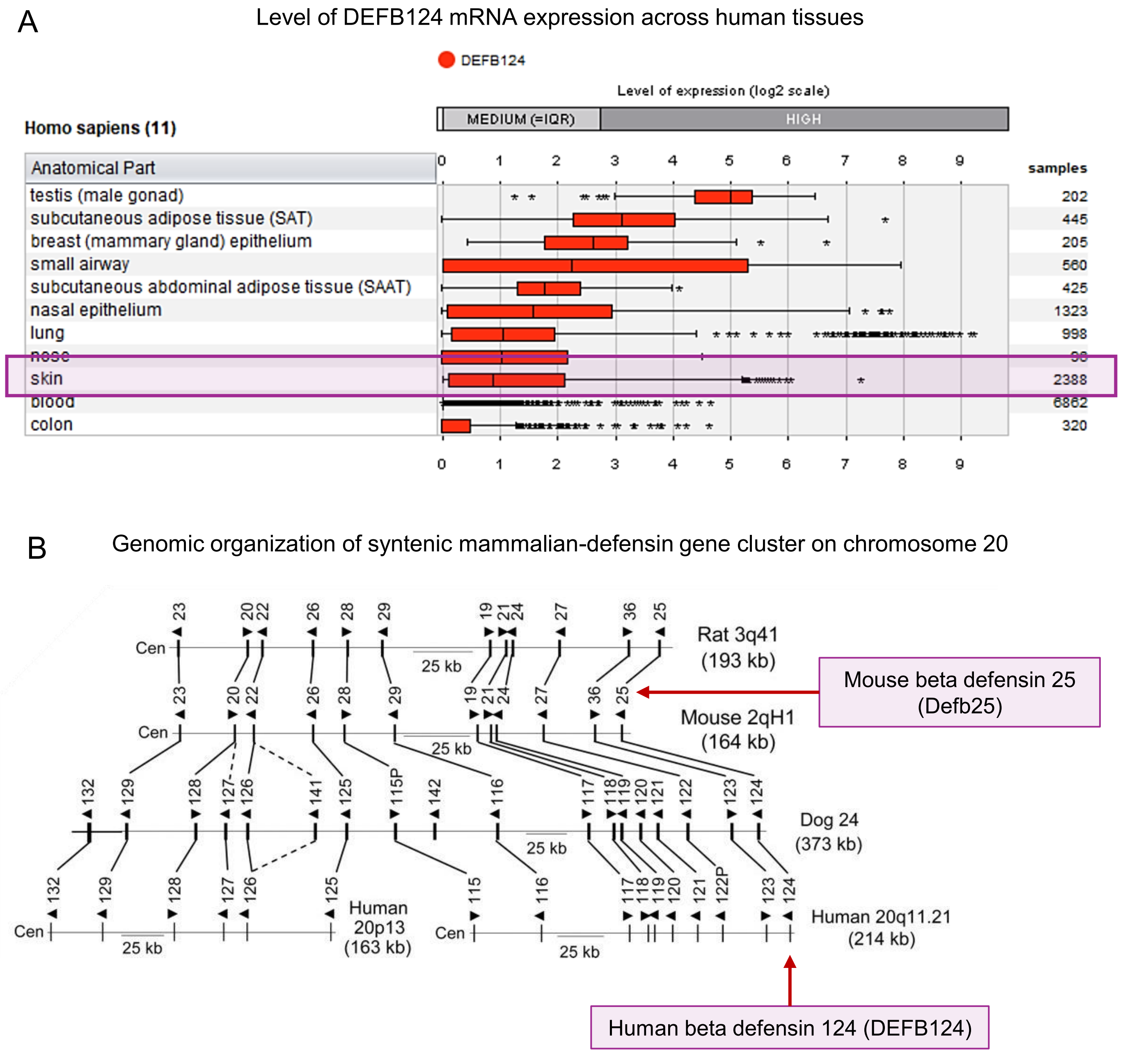

Figure S1. Expression pattern of human DEFB124 across tissues and comparative genomic organization with murine Defb25, related to Figure 1.

(A) Transcriptomic profiling of DEFB124 mRNA expression across multiple human tissues using publicly available datasets curated in Genevestigator from the Gene Expression Omnibus (GEO). Only tissue types represented by more than 200 independent samples were included to ensure robust comparisons. Expression levels are displayed on a log_2 expression scale, with each bar representing the distribution of DEFB124 transcript levels within a given tissue type. The number of samples analyzed for each tissue is indicated on the right. (B) Comparative genomic organization of the syntenic mammalian β-defensin gene cluster across several species, illustrating the evolutionary conservation of defensin loci. The diagram shows defensin gene arrangements and relative genomic distances within the clusters in human chromosome 20q11.21, mouse chromosome 2qH1, rat chromosome 3q41, and dog chromosome 24. Individual β-defensin genes are indicated by arrowheads, with approximate intergenic distances marked in kilobases. The position of human β-defensin 124 (DEFB124) within the human cluster is highlighted, alongside its murine ortholog β-defensin 25 (Defb25) located within the corresponding mouse defensin locus.

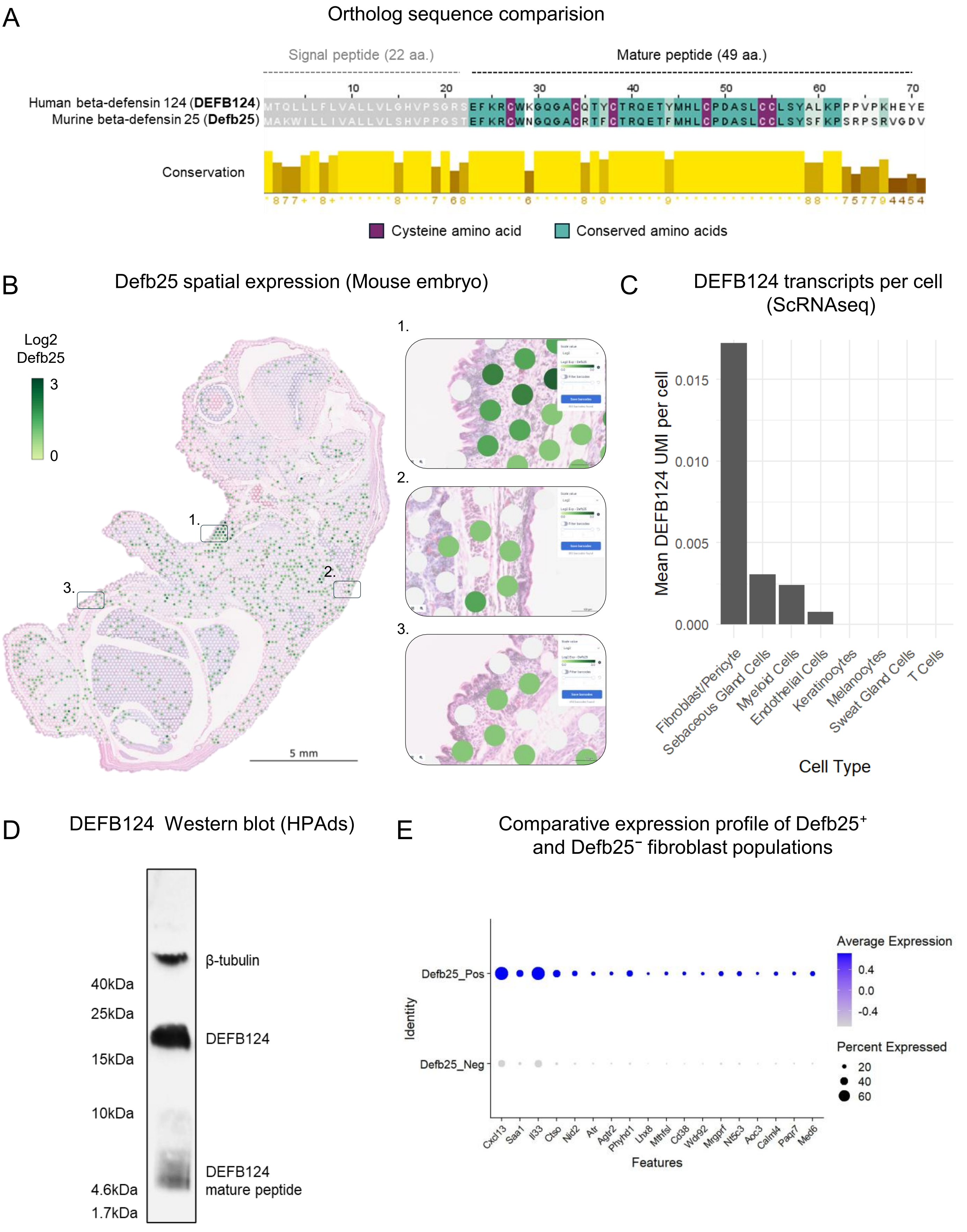

Figure S2. Cross-species sequence conservation and multi-level expression of DEFB124 and its murine ortholog Defb25, related to Figure 1.

(A) Orthologous protein sequence comparison between human β-defensin 124 (DEFB124) and murine β-defensin 25 (Defb25). The alignment highlights the signal peptide (22 amino acids) and the mature peptide (49 amino acids). Conserved amino acid residues are indicated in cyan, while cysteine residues characteristic of β-defensins are highlighted in purple. The conservation plot beneath the alignment shows residue-level conservation across homologous sequences, with higher scores indicating stronger evolutionary conservation between the two orthologs. (B) Spatial transcriptomic mapping of Defb25 expression in a mouse embryo.Transcript abundance is displayed on a log_2 scale, with green dots

Figure S2. Continued

indicating spatial spots containing detectable Defb25 transcripts. Insets (1–3) show higher magnification views of selected regions illustrating localized transcript signals within embryonic tissues. Scale bar, 5 mm. (C) Cell-type–specific expression of DEFB124 transcripts derived from single-cell RNA sequencing of human skin (dataset GSE147424). Mean DEFB124 UMI counts per cell are shown across major skin cell populations. Fibroblast/pericyte populations exhibit the highest transcript abundance, with lower levels detected in sebaceous gland cells, myeloid cells, and endothelial cells. (D) Western blot analysis of DEFB124 protein in human fibroblast like preadipocyte cell line (HPAds). (E) Dot plot showing relative expression of the top differentially expressed genes in Defb25-positive (Defb25_Pos) and Defb25-negative (Defb25_Neg) fibroblast populations identified from single-cell RNA-sequencing dataset shown in Figure 1C. Dot size represents the percentage of cells expressing each gene, while color intensity indicates average expression level. Defb25-positive fibroblasts demonstrate enrichment of inflammatory stromal-associated genes, including *Cxcl13*, *Saa1*, and *Il33*.

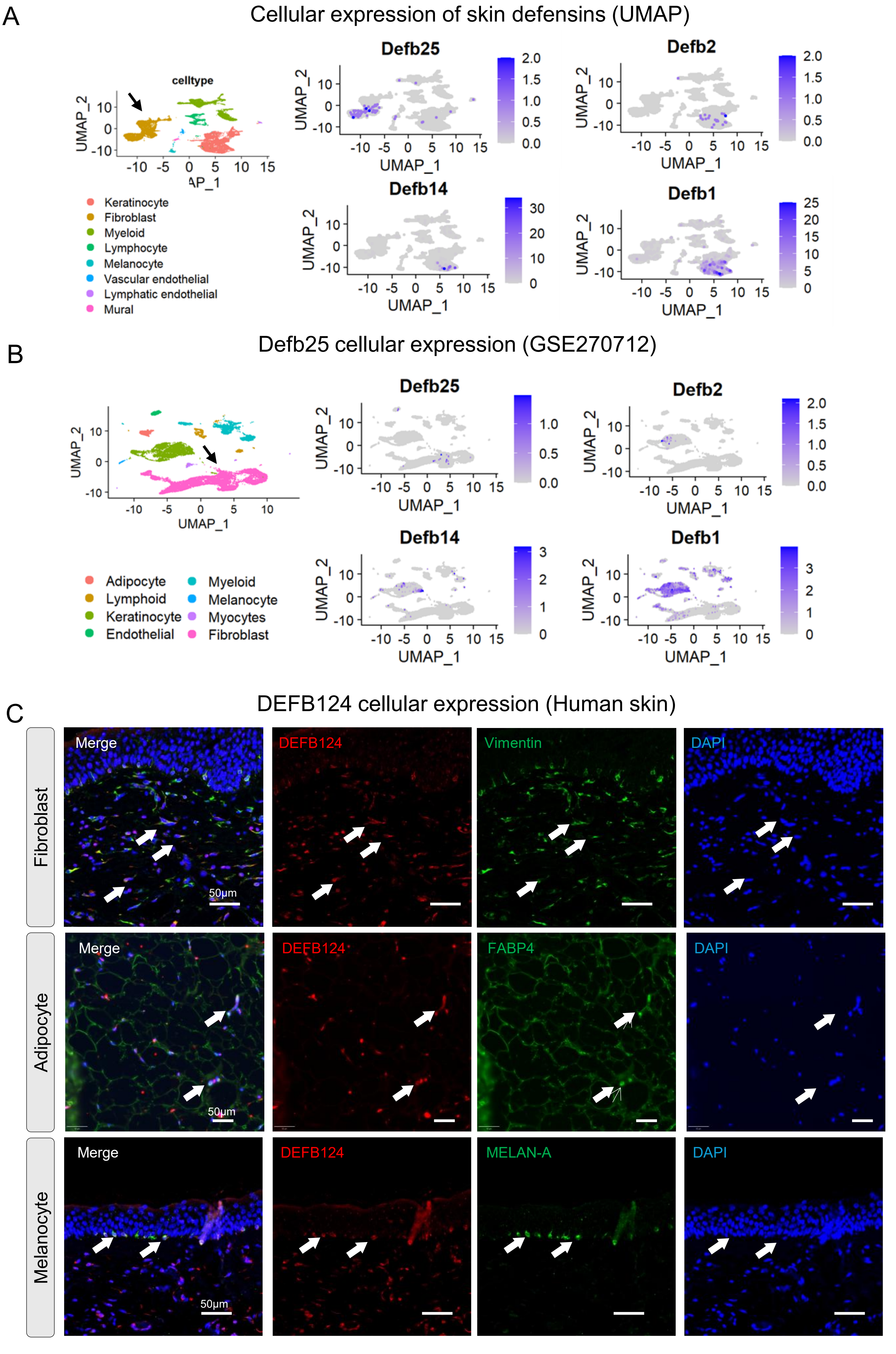

Figure S3. Stromal expression of defensin genes in mouse and human skin, related to Figure 1.

Figure S3. Continued

(A–B) Single-cell RNA sequencing analysis of defensin gene expression across mouse skin cell populations visualized by UMAP clustering from two independent datasets. Cell types were annotated based on transcriptional signatures. Feature plots show the distribution of Defb25, Defb2, Defb14, and Defb1 transcripts across clusters. In both datasets, Defb25 expression is primarily enriched in fibroblast populations (arrows), whereas other defensins display distinct expression patterns across epithelial, immune, and stromal cell types. Dataset shown in (B) corresponds to GSE270712. (C) Immunofluorescence staining of human skin sections showing cellular localization of DEFB124 protein. Panels display merged images and individual fluorescence channels for DEFB124 (red), cell-type–specific markers (green), and nuclei (DAPI, blue). Co-staining with vimentin identifies DEFB124 expression in dermal fibroblasts (top row). Additional staining with FABP4 (adipocyte marker) and MELAN-A (melanocyte marker) illustrates DEFB124 signal in adipocytes and melanocytes, respectively. Arrows indicate representative DEFB124-positive cells. Scale bars, 50 μm.

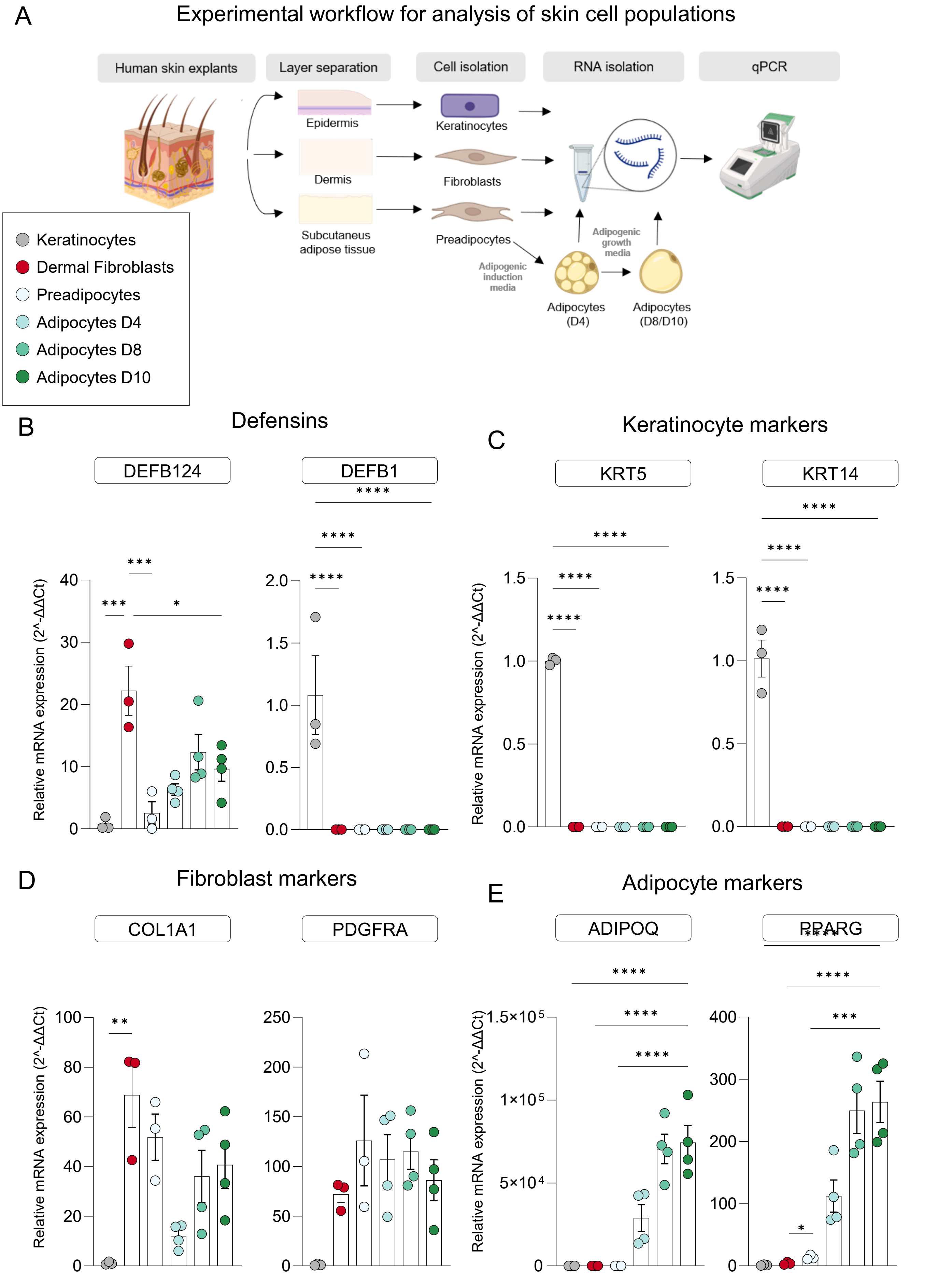

Figure S4: Expression profiling of DEFB124 across isolated human skin cell populations, related to Figure 1.

(A) Experimental workflow for isolation and characterization of human skin cell populations. Human skin explants were separated into epidermal, dermal, and subcutaneous adipose tissue fractions followed by isolation of keratinocytes, dermal

Figure S4. Continued

fibroblasts, and preadipocytes. Preadipocytes were differentiated into adipocytes and harvested at day 4 (D4), day 8 (D8), and day 10 (D10) for RNA isolation and qPCR analysis.(B) Relative mRNA expression of DEFB124 and DEFB1 across isolated human skin cell populations.(C) Relative mRNA expression of keratinocyte markers KRT5 and KRT14.(D) Relative mRNA expression of fibroblast markers COL1A1 and PDGFRA. (E) Relative mRNA expression of adipocyte markers ADIPOQ and PPARG during adipocyte differentiation. Gene expression was analyzed by qPCR and normalized to GAPDH expression using the 2^-ddCT method. Data are presented as mean ± SEM. Each dot represents an individual biological replicate. Abbreviations: D4, day 4; D8, day 8; D10, day 10. Statistical significance was determined using one way ANOVA ((n = 3–4 donor-derived biological replicates per group); *P < 0.05, **P < 0.01, ***P < 0.001, ****P < 0.0001

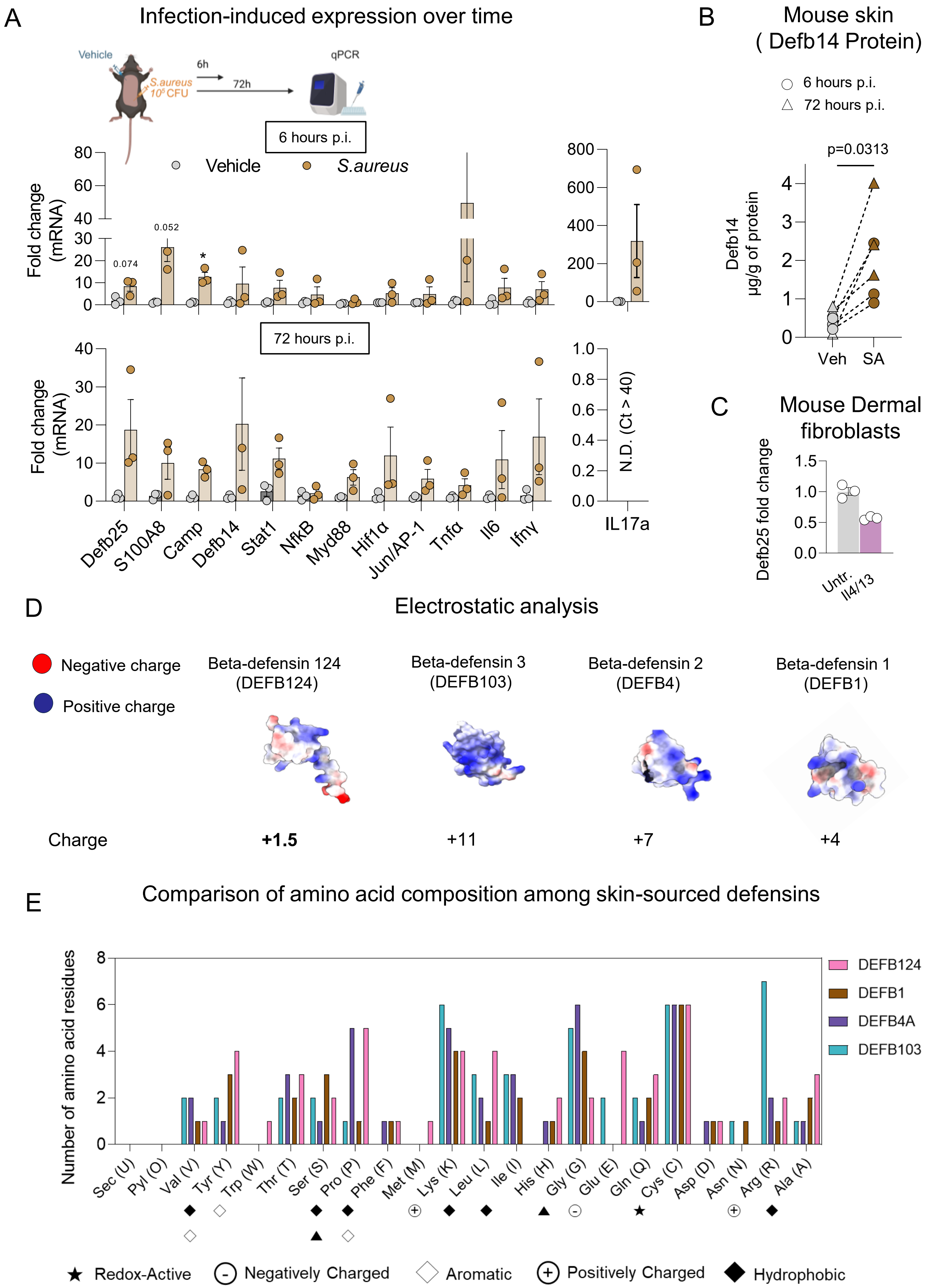

Figure S5. Regulation of Defb25 following *S. aureus* infection and structural comparison of skin defensins, related to Figure 2, Figure 3 and Figure 4.

(A) Time-course analysis of infection-induced gene expression in mouse skin. Mice were intradermally injected with *S. aureus* (10^6 CFU), and skin samples were collected at 6 h and 72 h p.i. for qPCR analysis. Expression levels of Defb25,

Figure S5. Continued

antimicrobial peptides, and inflammation-related genes are shown as fold change relative to vehicle-treated controls. Data illustrate induction of defensin and inflammatory response genes following infection. Infection with *S. aureus* increases spatial transcript signals for several defensin genes, including Defb25, within the skin. (B) Quantification of Defb14 protein levels in mouse skin following *S. aureus* infection. Measurements were performed at 6 hours and 72 hours post-infection (p.i.), showing increased Defb14 protein levels in infected skin compared with vehicle controls paired t-test, $p = 0.0313$; $n = 3$ per group per time point). (C) Defb25 mRNA expression in mouse dermal fibroblasts treated with Il-4 and Il-13. (D) Electrostatic surface analysis of β-defensin 124 (DEFB124) compared with other human β-defensins (DEFB103A, DEFB4A, and DEFB1). Structural models are colored according to electrostatic potential, with positive charge (blue) and negative charge (red). Net peptide charge is indicated below each structure. (E) Comparison of amino acid composition among skin-associated defensins (DEFB124, DEFB1, DEFB4A, and DEFB103). The bar graph shows the number of residues for each amino acid in the peptide sequences. Symbols below the x-axis indicate residue properties, including redox-active, negatively charged, aromatic, positively charged, and hydrophobic amino acids.

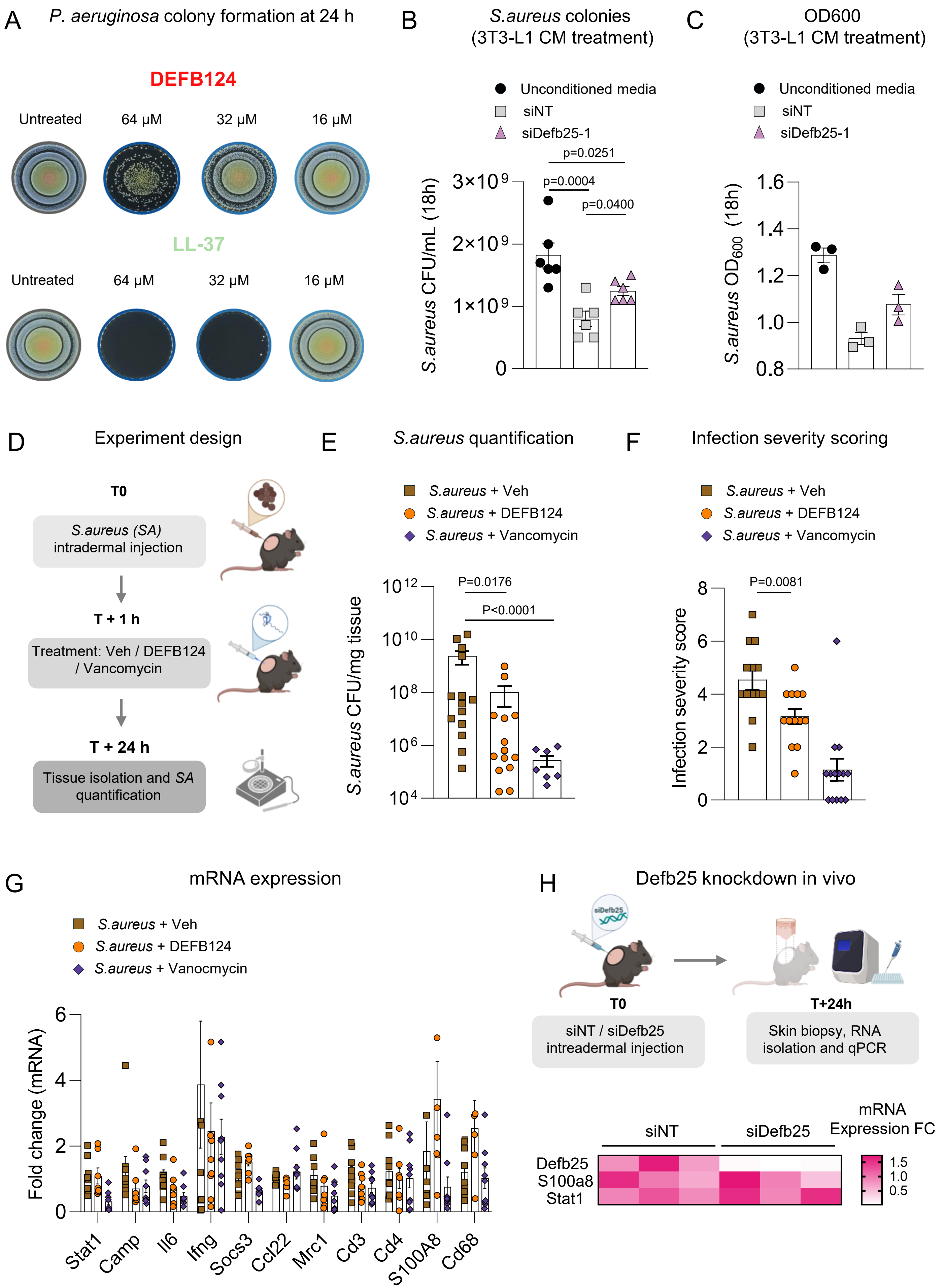

Figure S6. Antimicrobial and functional analysis of DEFB124/Defb25 against *S.aureus* infection, related to Figure 5, Figure 6 and Figure 7.

Figure S6. Continued

(A) Representative images of *P. aeruginosa* colony formation after 24 h under DEFB124 or LL-37 treatment. (B) *S.aureus* growth after incubation with fibroblast-conditioned media from unconditioned cultures, control siRNA (siNT), or siDefb25-treated fibroblasts, assessed by CFU at 18 h (B) and OD_{600} (C). (D) Relative mRNA expression of inflammatory and immune-response genes in infected skin, shown as fold change. (E) In vivo experimental design. Mice were intradermally injected with *S. aureus* (SA). One hour later, mice received intradermal treatment with vehicle, DEFB124 peptide, or vancomycin. Skin tissue was collected 24 h later for bacterial quantification. (F) Quantification of *S. aureus* burden in mouse skin expressed as CFU per mg tissue. DEFB124 treatment significantly reduced bacterial load compared with vehicle control, while vancomycin served as a positive antimicrobial control (DEFB124 vs vehicle, $p = 0.0176$; vancomycin vs vehicle, $p < 0.001$). $n = 9–14$ mice per group. Statistical analysis was performed using Kruskal–Wallis test followed by Dunn's multiple-comparisons test. Each point represents an individual mouse. (G) Infection severity scores in mice following intradermal *S. aureus* infection and treatment with vehicle, DEFB124, or vancomycin. (Mann–Whitney test), $n =$ 9–14 mice per group. (H) Validation of in vivo Defb25 knockdown following intradermal delivery of Defb25-targeting siRNA (siDefb25) or non-targeting control siRNA (siNT). Heatmap shows relative mRNA expression levels of Defb25 and inflammatory response genes in mice 24 h after siRNA injection. Each column represents an individual mouse. Data are shown as fold change (FC) relative to the siNT group.

AMP	Gene symbol	Fezakinumab	Gusacitinib (ASN002)	Crisaborole	Dupilumab (responders)	Ciclosporin (responders)
		Week 12, Microarray	Day 15, Microarray	Day 8 Microarray	Week 12, RNA-seq	Week 12, RNA-seq
Human β-defensin 124	DEFB124	1.76 (0.00141)	2.16 (0.086)	1.06 (ns)	3.1	2.4
Dermcidin	DCD	1.48 (0.066)	1.1 (ns)	1.35 (ns)	3.64	1.040067
Human β-defensin 1	DEFB1	1.02 (ns)	1.06 (ns)	-1.02 (ns)	1.323631	0.91
Ribonuclease 1	RNASE1	-1.19 (ns)	1.06 (ns)	1.12 (ns)	1.508682	0.895958
Ribonuclease 6	RNASE6	N.D.	-1.16 (ns)	1.27 (0.0181)	1.061036	0.756492
Cathelicidin	CAMP	N.D.	N:D.	-1.19 (0.008)	0.901312	0.338948
Human β-defensin 3	DEFB103	-1.66 (9.7×10^{-6})	-1.16 (ns)	.1.97 (1.83×10^{-7})	0.145724	0.134109
Ribonuclease 7	RNASE7	-1.68 (0.004)	0.597 (ns)	-1.76 (6.7×10^{-7})	0.95282	1.352182
Human β-defensin 2	DEFB4A	-10.95 (8.02×10^{-9})	-10.05 (0.0302)	-13.02 (2.19×10^{-10})	0.064999	0.073124
S100 calcium binding protein A8	S100A8	-1.79 (0.000265)	-2.26 (0.021)	-2.2 (2.4×10^{-6})	0.188116	0.246889

Table S1. Regulation of antimicrobial peptide (AMP) genes in skin biopsy transcriptomic datasets from clinical studies of atopic dermatitis (AD) therapies.

Fold-change values for selected AMP genes were extracted from previously published skin transcriptomic datasets of AD patients treated with dupilumab, crisaborole, gusacitinib (ASN002), fezakinumab, cerdulatinib, or ciclosporin. Expression changes are shown for the indicated treatment time points and platforms (microarray or RNA-seq). For datasets reporting fold regulation (microarray studies of fezakinumab, gusacitinib/ASN002, and crisaborole), positive values indicate increased expression and negative values indicate decreased expression relative to baseline; corresponding *p*-values are shown in parentheses where available. For datasets in which fold changes were not directly reported (dupilumab and ciclosporin), fold changes were calculated as the ratio of mean expression in treated samples relative to baseline lesional samples; values >1 indicate increased expression and values <1 indicate decreased expression. In these datasets, comparisons were performed between responders at 3 months post-treatment and baseline lesional samples. Because only aggregated expression values were available, statistical testing could not be performed for these datasets. N.D., not detected; ns, not significant.

Gene	Forward Primer (5'→3')	Reverse Primer (5'→3')
Il17a	TTTAACTCCCTTGGC GCAAAA	CTTTCCCTCCGCATT GACAC
Mrc1 (CD206)	CTCTGTTCAGCTATTGGACGC	CGGAATTTCTGGGATTCAGCTTC
Socs3	ATGGTCACCCACAGCAAGTTT	TCCAGTAGAATCCGCTCTCCT
Il22	CATTGCCTTCTAGGT CTCCTCA	CCTGCTTGCCAGTGC AAAAT
Stat6	CTCTGTGGGGCCTAATTTCCA	CATCTGAACCGACCAGGAACT
Ccl22	AGGTCCCTATGGTGCCAATGT	CGGCAGGATTTTGAGGTCCA
Tbp	CTTCCTGCCACAATGTCACAG	CCTTTCTCATGCTTGCTTCTCTG
Tnfα	CCCTCACACTCAGAT CATCTTCT	GCTACGACGTGGGCT ACAG
Gapdh	GGAGCGAGATCCCTCCAAAAT	GGCTGTTGTCATACTTCTCATGG
Defb25	AGTCATGTACCACCAGGAAG	GAATAGGACAGGCAACACAG
S100A8	AAATCACCATGCCCTCTACAA	CCCACTTTTATCACCATCGCA
Camp	GCTGTGGCGGTCACT ATCAC	TGTCTAGGGACTGCT GGTTGA
Il6	TGGGGCTCTTCAAAA GCTCC	AGGAACTATCACCGG ATCTTCAA
Cd3e	TCAGCCTCCTAGCTGTTGG	GTCAACTCTACACTGGTTCCTG
Cd4	CTAGCTGTCACTCAAGGGAAGA	CGAAGGCGAACCTCCTCTAA
Stat1	TCACAGTGGTTCGAG CTTCAG	GCAAACGAGACATCA TAGGCA
Stat3	CAATACCATTGACCT GCCGAT	GAGCGACTCAAACTG CCCT
Defb14	TCCAGGGGACGCATT CCTA	ACCGCTATTAGAACA TCGACCTA
Myd88	TCATGTTCTCCATAC CCTTGGT	AAACTGCGAGTGGGG TCAG
Hif1α	ACCTTCATCGGAAAC TCCAAAG	CTGTTAGGCTGGGAA AAGTTAGG
Jun&AP-1	CCTTCTACGACGATG CCCTC	GGTTCAAGGTCATGC TCTGTTT
NfKb1	ATGGCAGACGATGAT CCCTAC	TGTTGACAGTGGTAT TTCTGGTG
Ifng	ATGAACGCTACACACTGCATC	CCATCCTTTTGCCAGTTCCTC
Cd68	TGTCTGATCTTGCTAGGACCG	GAGAGTAACGGCCTTTTTGTGA
DEFB124	CCTTCCAAGAGAGGCGGATT	TCCAAGCCACCTCCCAAAAT
KRT14	TGAGCCGCATTCTGAACGAG	GATGACTGCGATCCAGAGGA
KRT5	TGACCTCCGCAACACCAAG	CAGATTGGCGCACTGTTTCTT
COL1A1	GAGGGCCAAGACGAAGACATC	CAGATCACGTCATCGCACAAC
PDGFRA	TTTTTGTGACGGTCTTGGAAGT	TGTCTGAGTGTGGTTGTAATAGC
MITF	GCCTGTCTCGGGAAACTTGA	TCTTGCTTCAGACTCTGTGGG
	Catalogue number	
ADIPOQ	Hs00605917_m1	
PPARg	Hs01115513_m1	
PLIN1	Hs00160173_m1	
FABP4	Hs01086177_m1	

Table S2. qPCR primers used in the study

	Final concentration in medium	Stock solution
DMEM/F-12, Glutamax, HEPES	1 x	-
Fetal Bovine Serum (FBS)	5%	-
rh EGF	10 ng/ml	100 µg/mL in 0.1% BSA
Heparin	90 µg/ml	9 mg/ml DMEMF12
Hydrocortisone	1 µg/ml	1 mg/mL Ethanol absolut
Insulin	0.5 µg/ml	-
Primocin	100 µg/ml	-

Table S3. Composition of preadipocyte cultivation medium including concentrations of stock solutions.

Adipogenic Induction Medium		
	Final concentration in medium	Stock solution
D-Pantothenic acid	15 µM	15 mM in DMEM/F-12
Biotin	33 µM	33 mM in DMEM/F-12
ApoTransferrin	10 µg/ml	10 mg/ml in DMEM/F-12
Insulin	0.1 µM	10 mg/ml or 1.744 mM
Dexamethasone	0.25 µM	500 µM in Ethanol abs.
Rosiglitazone	1 µM	700 µM in Ethanol abs.
IBMX	500 µM	250 mM in DMSO
Penicillin-Streptomycin	1%	10000 U/ml

Table S4. Composition of adipogenic induction medium.

Adipocyte Growth Medium		
	Final concentration in medium	Stock solution
D-Pantothenic acid	15 µM	15 mM in DMEM/F-12
Biotin	33 µM	33 mM in DMEM/F-12
ApoTransferrin	10 µg/ml	10 mg/ml in DMEM/F-12
Insulin	0.01 µM	10 mg/ml or 1.744 mM
Penicillin-Streptomycin	1 %	10000 U/ml

Table S5. Composition of adipocyte growth medium.

Collection Date	Sex	Birth Year	BMI	Age
08.07.2021	Female	1985	28.9	36
13.07.2021	Female	1982	27.0	39
05.11.2021	Female	1990	26.8	31
06.07.2022	Female	1989	24.8	33

Table S6. Donor Characteristics of Human preadipocytes

Collection Date	Sex	Birth Year	BMI	Age
19.06.2023	Female	1984	–	39
21.06.2023	Male	1992	–	31
21.08.2023	Female	1981	–	42

Table S7. Donor characteristics of dermal fibroblasts, and keratinocyte samples
